# Rehabilitation after bone marrow transplant compared with usual care to improve patient outcomes (REBOOT): protocol for a randomised controlled trial

**DOI:** 10.1186/s12885-025-13898-3

**Published:** 2025-03-24

**Authors:** Linda Denehy, Shaza Abo, Christopher Swain, Camille E. Short, Nicole Kiss, Amit Khot, Eric Wong, Duncan Purtill, Clare O’Donnell, Marlena Klaic, Catherine L. Granger, Michelle Tew, Tim Spelman, Vinicius Cavalheri, Lara Edbrooke, on behalf on the REBOOT research team, Ailish Doyle, Anna Beaumont, Annaliese Mackie, Belinda Herrmann, Bianca Ukovic, Claire McRae, Danika Carty, Emily Calton, Fiona Coll, Gerald Yeo, Jazmin Brooks, Joanne Houston, Kate Kaegi, Kristen Capron, Lucy Troup, Maddie Adair, Michelle Kendell, Molly Whitaker, Morgan Smith, Paul Gittings, Rachel McLean, Rebekah Dempsey, Rhyan Fry, Sangeeta Sathyanath, Stacey Haughton, Stephanie Versaci, Talia Clohessy, Thomas Phyland

**Affiliations:** 1 Department of Physiotherapy, The University of Melbourne, 161 Barry Street, Parkville, VIC 3010 Australia; 2 Department of Health Services Research, Peter Maccallum Cancer Centre, 305 Grattan Street, Melbourne, VIC 3000 Australia; 3 Department of Oncology, Sir Peter Maccallum, University of Melbourne, Parkville, VIC 3010 Australia; 4 Department of Physiotherapy, Peter MacCallum Cancer Centre, 305 Grattan Street, Melbourne, VIC 3000 Australia; 5 Melbourne Centre for Behaviour Change, The University of Melbourne, 800 Swanston St, Melbourne, VIC 3053 Australia; 6 Institute for Physical Activity and Nutrition, Deakin University, Burwood, VIC 3125 Australia; 7 Clinical Haematology, Peter Maccallum Cancer Centre and Royal Melbourne Hospital, Melbourne, Australia; 8 Clinical Haematology Austin Health, 145 Studley Road, Heidelberg, VIC 3084 Australia; 9 Department of Haematology, Fiona Stanley Hospital, Perth, WA Australia; 10 Department of Haematology, Pathwest Laboratory Medicine, Perth, WA Australia; 11 Department of Physiotherapy, Austin Hospital, 145 Studley Road, Heidelberg, VIC 3084 Australia; 12 Melbourne School of Health Sciences, the University of Melbourne, 161 Barry Street, Parkville, VIC 3010 Australia; 13 Melbourne Health Economics, Centre for Health Policy, Melbourne School of Population and Global Health, University of Melbourne, Parkville, VIC 3010 Australia; 14 Faculty of Health Sciences, Curtin School of Allied Health, Curtin University, GPO Box U1987, Perth, WA 6845 Australia; 15 Allied Health, South Metropolitan Health Service, 11 Robin Warren Drive, Murdoch, WA 6150 Australia

**Keywords:** Rehabilitation, Haematology, Cancer, Bone marrow transplant, Stem cell transplant, Exercise, Nutrition, Telehealth, Behaviour change, Physical function

## Abstract

**Background:**

Haematological cancer affects more than 1.3 million people around the world annually and accounted for almost 800,000 deaths globally in 2020. The number of patients with these cancers undergoing bone marrow transplant is increasing. Of note, this intensive treatment is associated with complex and multifactorial side effects, often impacting nutritional status, physical functioning and overall health-related quality of life. The primary aim of this study is to investigate the effectiveness of an eight-week multidisciplinary rehabilitation intervention compared with usual care on the physical function domain of the European Organisation for the Research and Treatment of Cancer quality of life questionnaire (EORTC QLQ-C30 version 3) in patients with haematological cancer following bone marrow transplant.

**Methods:**

This is a multisite, pragmatic two-arm parallel-group, randomised controlled trial (RCT) with stratified randomisation, powered for superiority, recruiting 170 participants at 30 days following either allogeneic or autologous bone marrow transplant (ACTRN12622001071718). Recruitment sites include three Australian university affiliated teaching hospitals. Participants are eligible if aged ≥ 18 years, treated for haematological cancer with allogeneic or autologous bone marrow transplant and can walk independently. The intervention group will receive eight weeks of twice weekly telehealth-based exercise classes, an initial and follow up dietetics consult, post exercise protein supplements, and a home-based physical activity program, all with embedded behaviour change strategies. The primary outcome is patient reported physical function measured using the EORTC QLQ-C30 version 3. Secondary outcomes include other domains of the EORTC QLQ-C30, fatigue, physical function, physical activity levels, frailty, body composition, sarcopenia and nutrition assessment. We will also undertake a health economic analysis alongside the trial and a process evaluation exploring intervention fidelity, causal mechanisms as well as contextual influences through qualitative enquiry.

**Discussion:**

The REBOOT trial will add RCT-evidence from a rigorously conducted, statistically powered multi-site trial to existing limited knowledge on the effects of multi-disciplinary rehabilitation for people with haematological cancer. If effectiveness is supported, then implementation of rehabilitation into care pathways for people having bone marrow transplant can be considered.

**Trial registration:**

ACTRN12622001071718 prospectively registered 03/08/2022, last updated 08/03/2024.

**Supplementary Information:**

The online version contains supplementary material available at 10.1186/s12885-025-13898-3.

## Background

Haematological cancer affects more than 1.3 million people around the world annually and accounted for almost 800,000 deaths globally in 2020 [1, 2]. In the US more than 180,000 new haematological cancer cases (leukaemia, myeloma or lymphoma) were predicted in 2023, with approximately 1.6 million people living with a haematological cancer [3]. Similarly, in Australia this incidence has risen by nearly 50% over the past 10 years and expected to rise further by 2035 [4, 5]. Haematopoietic stem cell transplantation (HSCT), also often called bone marrow transplantation (BMT, the term used throughout this paper), is a treatment that is commonly used for patients with haematological cancers in an attempt to achieve long-term disease response. Treatment for haematological cancer with BMT is increasing by an estimated 7% annually worldwide [6]. However, the intensity of this treatment results in considerable adverse effects including deleterious impacts on nutritional status, physical functioning and overall health-related quality of life [7, 8].

Compelling evidence from large scale randomised controlled trials (RCTs) demonstrates that tailored exercise interventions can improve symptoms associated with cancer treatment, including health related quality of life (HRQoL), fatigue and physical functioning [9], however the majority of this research comes from solid tumours. There is limited high-quality research in haematological cancers or in BMT. Time, treatment sequalae and recovery following BMT vary depending on the underlying haematological cancer (leukaemia, lymphoma or myeloma), conditioning therapy, the type and intensity of transplant, and patient factors [10]. A recent meta-analysis reports exercise interventions for people treated with BMT are effective at improving multiple factors of physical and psychosocial health including functional exercise capacity, global HRQoL and fatigue. However, conclusions are limited by small sample sizes (median [IQR] 42 [30–100] participants), and flawed study designs, for example inadequate assessor blinding and concealment, introducing risk of detection and selection bias [11].

Multimodal cancer care should include nutrition support as a vital component due to the significant impact of cancer-related malnutrition, which remains underestimated and undertreated worldwide [12]. Nutrition support may be available during BMT inpatient hospital admission, however, severe early (e.g., mucositis) and late (e.g., graft-versus-host-disease) gastrointestinal toxicities may prohibit adequate nutritional intake, meaning targeting nutrition rehabilitation following discharge is an important goal. Most nutrition research in people undergoing BMT has focused on interventions to minimise loss of muscle mass during the BMT admission [13]. Few studies have included nutrition as a component of multimodal rehabilitation interventions after the BMT admission, and those that have included nutrition tend to focus recommendations on reduction of longer-term cardio-metabolic risk [14]. It is well known that diet and physical activity are essential to healthy lifestyles, and growing evidence in solid tumours suggests that nutrition counselling and whey protein supplementation alongside exercise training can lead to clinically meaningful improvements in functional exercise capacity [15, 16].

To support patients with cancer to adopt and sustain healthy exercise and nutrition behaviours, behaviour change support is essential [17, 18]. Best practice involves considering key modifiable influences on behaviour and mapping appropriate behaviour change support to address them [18, 19]. In this setting, behaviour change techniques to support patients with motivation and self-efficacy, whilst working within the constraints of physical and mental health issues are especially needed [20–22].

Despite the importance of physical activity, exercise, nutrition and behaviour change support for patients to recover after a BMT, rehabilitation programs incorporating these strategies following BMT treatment are not routinely provided in clinical practice globally [23, 24] possibly related to the lack of a robust body of evidence supporting such programs.

In order to increase equity and access to patients living in regional areas, and those with work and carer responsibilities who face significant barriers to accessing in-clinic sessions [25, 26], models of rehabilitation should also consider use of tele-health. There is consistent evidence that cancer survivors prefer flexible home-based programs [27]. Feedback specifically from people treated with BMT report that limited accessibility to exercise programs is a barrier, whilst flexibility, individualisation, socialisation from a group-based approach and education are important facilitators to participation in rehabilitation [28]. Technological advances make it increasingly possible to embed intervention components including individualised nutrition support, supervised tailored exercise prescription, behaviour change and social support [29] into home-based programs. Recent evaluations in other clinical populations suggest these interventions are equally effective and demonstrate similar adherence and satisfaction compared to standard in-clinic rehabilitation programs [30]. Use of telehealth provides an opportunity to address health inequity, improve health outcomes for patients, and offers an alternative, particularly among immunosuppressed individuals.

The REhabilitation after BOne marrOw Transplant to improve patient outcomes (REBOOT) trial is a multi-site randomised controlled trial using a remote intervention that is adequately powered to test the effectiveness of multidisciplinary tele-rehabilitation including nutrition, exercise, physical activity and behaviour change following allogeneic or autologous BMT. These are crucial factors, as previous research has been underpowered or used unimodal interventions. The overarching aim of the REBOOT trial is to investigate the effectiveness of an eight-week multidisciplinary rehabilitation intervention (nutrition, exercise, physical activity and behaviour change) compared with usual care on both clinical and patient reported outcomes.

## Methods

**Fig. 1:**
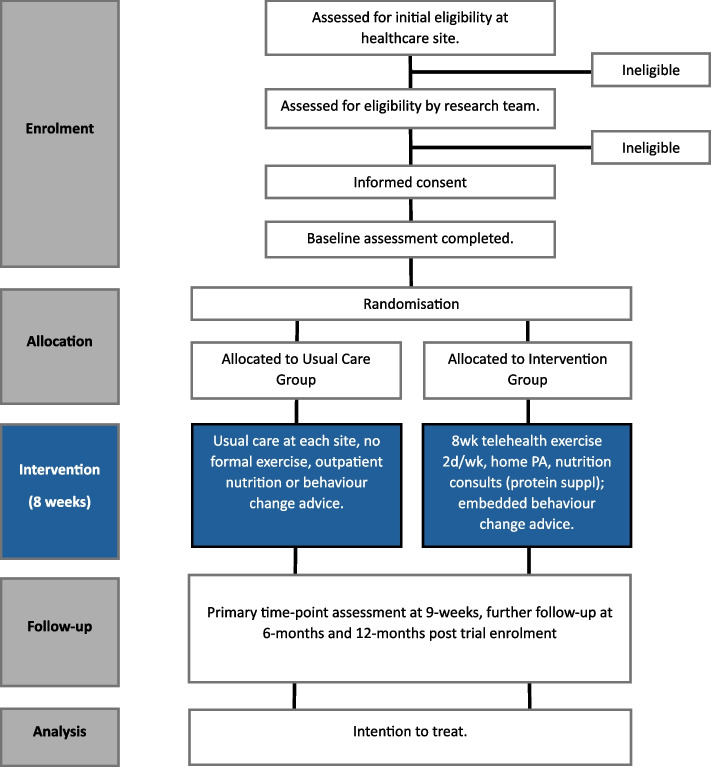
Flow diagram of the trial protocol. A flow diagram developed according to CONSORT guidelines [32, 33], describing the enrolment, randomization, follow-up, and analysis of the trial. *Abbreviations: PA,* physical activity

This protocol is reported according to the Standard Protocol Items: Recommendations for Interventional Trials (SPIRIT) 2022 guidelines [31]. Figure 1 outlines the planned participant flow through the trial using the Consolidated Standards of Reporting Trials [32, 33].

### Study aims

#### Primary aim

To investigate the effectiveness of an eight-week multidisciplinary rehabilitation intervention (nutrition, exercise, and behaviour change) on the physical function domain of the European Organisation for the Research and Treatment of Cancer (EORTC QLQ-C30 version 3 [34]) health-related quality of life (HRQoL) questionnaire in patients with haematological cancer following BMT (allogeneic or autologous), compared to usual care.

#### Primary hypothesis

The intervention will be effective in improving self-reported physical function at nine weeks post recruitment compared to usual care.

#### Secondary aims

To investigate the effectiveness of the intervention, including measures of 1) physical activity levels and objective physical function, 2) nutritional status, clinical frailty, sarcopenia, and body composition, 3) muscle strength, 4) fatigue, 5) health service use and costs, 6) global HRQoL and 7) survival.

Further, exploratory aims of this trial are

1. to determine the impact of treatment on rectus femoris muscle cross sectional area and echogenicity (muscle quality) using point of care muscle ultrasound in a sub-set of participants at one centre,
2. to examine the impact on outcomes in a subset of participants who received both prehabilitation (as part of usual care at one centre) and the trial rehabilitation intervention,
3. to conduct a process evaluation exploring fidelity, causal pathways and contextual influences.

### Study design and setting

**Fig. 2:**
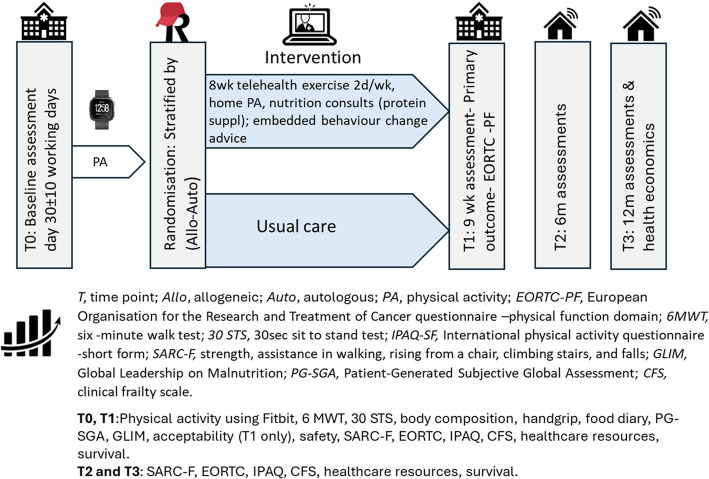
Diagram of trial design and outcomes

The REBOOT trial is a multi-site parallel group, two-arm, randomised controlled superiority trial in patients with haematological cancer who are 30 ± 10 working days following either allogeneic or autologous BMT (Fig. 2). Participants will be recruited from three Australian government-funded, metropolitan, quaternary, university affiliated, teaching hospitals: Peter MacCallum Cancer Centre (Melbourne, Victoria), Austin Health (Melbourne, Victoria) and Fiona Stanley Hospital (Perth, Western Australia). One trial site performs only autologous transplants (Peter MacCallum Cancer Centre) whilst two sites perform both allogeneic and autologous transplants. Data will be collected within hospital outpatient clinics and online. The intervention will be delivered remotely via videoconferencing.

### Participants

Eligibility criteria are detailed in Table 1. In brief, adults aged ≥ 18 years, treated for haematological cancer with allogeneic or autologous BMT and who can walk independently will be considered for participation in the trial at 30 ± 10 working days following transplant. Any cases where eligibility is unclear will be discussed with the Chief Investigator and/or senior clinical team prior to recruitment.

**Table 1:** Participant eligibility criteria

Inclusion Criteria	Exclusion criteria
• 30 ± 10 working days after allogeneic or autologous stem cell transplant for the treatment of haematological cancer	• Concurrent, actively treated other malignancy or history of other malignancy treated within the past year;
• ≥ 18 years of age	• Severe or unstable neurological, cardiorespiratory or musculoskeletal disease or mental illness that might compromise ability to perform exercise
• Ambulating independently	• Unstable psychiatric or cognitive disorders
• Proficient in English to understand testing and training	• Eastern Cooperative Oncology Group (ECOG) performance status > 2

### Provider inclusion criteria

The exercise intervention will be delivered by physiotherapists who are ≥ 2 years since graduation and have experience in managing patients with cancer. The nutrition intervention will be delivered by dietitians experienced in cancer management. The behaviour change component will be included within exercise and nutrition interventions with training for all intervention staff from a behaviour change scientist. All intervention staff will be trained in providing telehealth-based exercise classes using on-line recorded videos as well as face to face demonstration and intervention manuals. Outcome assessors will be physiotherapists and dietitians who are blinded to the group allocation, trained in all outcomes and provided with a comprehensive training manual. Training of all new staff will be documented and training updates for staff will occur each year.

### Recruitment and informed consent

The list of people receiving stem cell transplantation at each hospital will be screened weekly by a member of the research team for patient eligibility. Weekly discussions between the nurse and trial co-ordinator as well as within the relevant multidisciplinary meetings will support recruitment to the trial. Potential participants meeting eligibility will receive a phone call (or face to face discussion if patient is in hospital) from a member of the clinical research team to assess interest in the trial. Those who express an interest will be provided with an information flyer and patient information and consent form (PICF), which will be explained in more detail at this time and the individual will be formally invited to participate. At the initial clinic visit, the member of the research team will reiterate the aims, methods (including use of telehealth review consultations), potential benefits and hazards, confirm understanding of the trial and the PICF, allow an opportunity to ask questions, and obtain written informed consent from participants prior to commencing any procedures or assessments. The consent form will be signed by the patient with the date and time of that signature indicated. The Investigator(s) will keep the original consent forms and copies will be given to the patient. Enrolment to the trial will be documented in the patient’s electronic medical record. Written informed consent will be gained either (1) via electronic signature on the PICF using the Research Electronic Data Capture web-based application (REDCap database); or (2) via manual signature on a paper copy of the PICF. Paper records will be kept in a secure filing cabinet at each site.

### Randomisation

Following completion of baseline testing, participants will be randomly allocated (1:1) to either the usual care or exercise and nutrition group. Randomisation will be generated by an independent statistician not involved in the trial using permuted random blocks with stratification according to BMT type (allogeneic or autologous). Randomisation will be concealed using the REDCap randomisation module and performed by a researcher not involved in recruitment or trial measurements.

### Blinding

All post randomisation outcome assessments will be undertaken by assessors blinded to group assignment, but it is not possible to blind staff delivering the intervention or participants due to the nature of the intervention. The study statistician and health economist will also be blinded to group assignment to undertake analyses.

### Study interventions

#### Usual care

Both groups will receive usual medical and nursing care after transplant, which does not routinely involve exercise or nutritional interventions beyond the inpatient period. At the recruitment hospitals (consistent with broad Australian practice) at the time of trial commencement there was no rehabilitation model of care offered after transplant and therefore this comparator was appropriate. Participants will receive usual care face to face and printed educational materials about BMT from nursing and medical staff. These have been developed specifically by the recruitment hospitals and are part of usual care. At one centre prehabilitation is already part of usual care and will be provided according to the hospital protocols in the four to six weeks prior to transplant based upon individual assessment. This includes aerobic and resistance exercise sessions and may include dietary advice and psychological support. The details of these sessions as part of usual care are recorded in the electronic medical record. Participants receiving both prehabilitation and the REBOOT trial rehabilitation intervention will form a subgroup for analysis. All participant usual care will be documented.

### Intervention

The intervention is an eight-week, multidisciplinary exercise, physical activity, nutrition, and embedded behaviour change program that will be provided in addition to usual care. The goal of the intervention is to improve aerobic fitness, muscle mass, nutritional status and strength and facilitate increased physical activity at home following transplant. Following randomisation, participants allocated to the intervention group will be provided with an intervention pack including three different coloured resistance bands (light, moderate, firm resistance), a “Physical Activity and Nourishing Eating Toolkit” (consumer-reviewed, evidence-based education booklet regarding exercise and physical activity, nutrition and behaviour change specifically developed for this trial), a laminated goal-setting calendar, and protein supplement, and be asked to continue wearing their Fitbit activity monitor. Exercise intervention is reported according to the Consensus on Exercise Reporting Template (CERT, Supplementary Table 1) [35].

#### Exercise intervention

For the exercise component, participants will complete exercise sessions, lasting 60 to 75 min, supervised by a physiotherapist, twice weekly for eight-weeks delivered via telehealth. There will be rolling recruitment to the trial with exercise sessions group based where possible with up to four participants (if more than four then extra sessions will be run).

The delivery model will be via telehealth using videoconferencing platforms available within recruitment sites and/or the Zoom platform. The baseline session will be face to face to undertake measurements and teach use of the Fitbit activity monitor. Additionally, participants in the intervention group will be offered a face to face session or a one-on-one telehealth session with the physiotherapist at approximately 4 weeks to assess that exercises are being performed optimally. Intervention exercise sessions will commence at the next available session after the initial nutrition intervention session and within two weeks of baseline assessment (30 days ± 10 working days post-BMT). Participants attending their first exercise intervention session will join the telehealth session 15 min prior to the other participants in the group for one-to-one education with the physiotherapist regarding exercise in haematological cancer, the traffic light screening and modified BORG rating of perceived exertion (RPE), use of the Zoom platform and prescription of an initial individualised home-based program at the participant’s current physical functioning level.

Each session will include aerobic and resistance exercises, performed with resistance bands and using body weight. Exercises will be performed in intervals of four exercises per set and for three sets with approximately one minute of work followed by 30–60 s of rest. These will be individualised by the intervention physiotherapist depending upon participant ability and according to international exercise guidelines [36]. Aerobic interval training will take approximately 30 min and resistance training approximately 20 –25 min. Aerobic exercises will include for example, marching on the spot, (modified) star jumps, marching with concurrent upper limb exercises. Resistance exercises will include functional exercises such as sit to stand, heel-raises, squats, wall push ups and/or resistance band exercises such as shoulder flexion, abduction and elbow flexion (Table 2).

**Table 2:** Exercise Frequency, Intensity, Time, Type, Volume and Progression (FITT-VP) principles used for REBOOT intervention participants

	**Frequency**	**Intensity**	**Time**	**Type**	**Volume**	**Progression**
Aerobic training	2 days supervised on tele-health (3–5 days independent home program below)	Moderate to high – modified BORG RPE 4–6/10^a^	Interval training: 60 s of work, 30–60 s’ rest; 3 rounds of 4 exercises	Group-based, supervised. Functional, interval training, within home environment no equipment e.g., walking/jogging on the spot, star jumps	30 min twice/wk	Using BORG RPE ± HR from Fitbit during exercise sets: (HR max = 220-age)
Resistance training	2 days supervised on tele-health	Moderate to high – modified BORG RPE 4–6/10^a^	Interval training: 60 s of work, 30–60 s’ rest; 3 rounds of 4 exercises	Group-based, supervised. Functional, interval training, body weight and/or resistance bands, e.g., squats, wall push-ups	20 min twice/wk	
Home based program	3–5 days/ week independent, unsupervised	Moderate to high – modified BORG RPE 4–6/10	Suitable to participant	Aerobic only – e.g., walking, cycling program	Build up to ≥ 30 min daily	Goals ± review of steps (Fitbit)

^a^During the supervised tele-health program, intensity will be monitored using self-reported modified BORG RPE and Heart Rate (using Fitbit) after each exercise set, progressions adjusted accordingly. *Abbreviations: BORG RPE* modified BORG rating of perceived exertion scale, *HR* heart rate

**Fig. 3:**
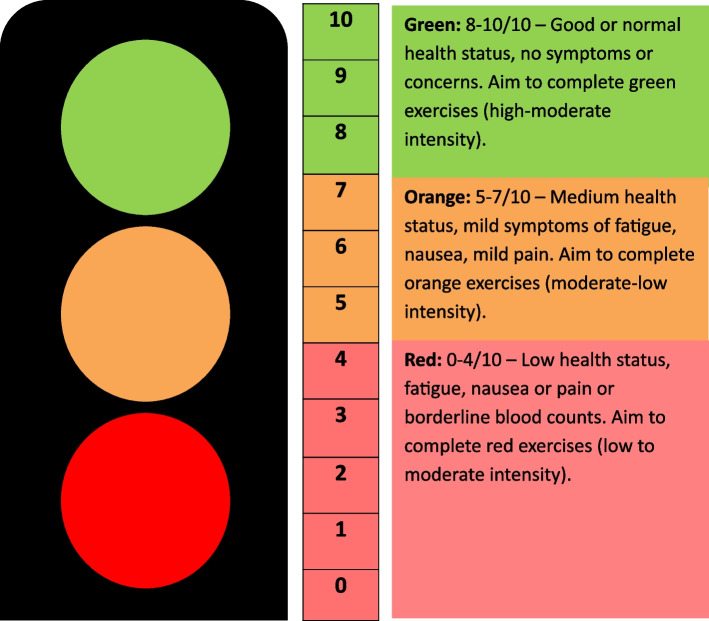
Traffic light system for participants to self-rate their wellness at the beginning of each exercise intervention session [37]. Physiotherapists will use this to help guide the intensity of prescribed exercises each session. Participants receive this scale as part of their “Physical Activity and Nourishing Eating Toolkit”

A traffic light screening system (Fig. 3 [37]) will be used before each session to gauge how participants are feeling that day. This will be used to guide exercise intensity and timing of intervals for that individual in the session. Heart rate (measured using a Fitbit) and/or modified BORG RPE will guide exercise intensity, aiming for a moderate-high intensity 4–6/10, and exercise progressed according to current exercise guidelines and participant ability [36]. Resistance exercises will be prescribed from baseline assessment findings and resistance band colour adapted using clinical expertise and progressed using modified BORG RPE.

A home program of moderate intensity continuous physical activity will be prescribed aiming to be active on the days not attending group sessions on telehealth. This program will be individualised, following the Frequency, Intensity, Time and Type, Volume and Progression (FITT-VP) principles (Table 2), and aim to achieve 3–5 days per week of moderate intensity walking (modified BORG 4–6/10). A Fitbit Charge 5 activity monitor will be provided to intervention participants to track their physical activity levels during the trial and may guide home program goals. Home program goals will be discussed with participants at each group supervised exercise session.

Participants will be reminded about their diet and importance of taking their protein supplementation within 30 min after each exercise session and adherence to this will be recorded by the physiotherapist.

### Nutrition

Prior to commencement of the exercise program and at 4-weeks, intervention participants will attend an individual 60-min session with the trial dietitian (telehealth or face to face). During this session, a comprehensive nutrition assessment will be completed to inform individualised dietary advice to achieve a protein intake of 1.5 g/kg body weight, and 25 to 30 kcal/kg/body weight aimed at optimising lean mass and preventing increase in fat mass. While a food-first based approach will be utilised to achieve protein and energy goals, participants will also be instructed to take a powdered whey-based protein supplement (20 g protein) within 30 min of exercise twice weekly, added to a liquid of their choice to help to preserve and build muscle mass. Dietary advice will be individualised according to participants nutritional status, physical function, symptoms, and food preferences and will address long-term treatment-related cardiometabolic consequences in addition to preserving muscle mass.

### Criteria for discontinuing or modifying allocated interventions

Prior to the commencement of exercise, physiotherapists will discuss any precautions and participants will be educated on safety criteria which will also be provided in written form in their “Physical Activity and Nourishing Eating Toolkit”. Participants will be asked about safety criteria including fever, pain, chest pain or any new changes. Exercise intervention will be modified according to the participant’s self-reported wellbeing using the traffic light screening system (Fig. 3). During the class, exercise intensity or timing will be modified by the physiotherapist if participants are breathless, working at a high BORG rate (9–10/10), approaching their HR-max according to Fitbit measurements, or otherwise feel unwell. Physiotherapists are trained in more detailed safety criteria and any adverse events are recorded and reported to the Chief Investigator [37].

The dietitian will monitor tolerance to the prescribed whey protein and prescribe an alternative protein supplement if needed.

### Strategies to improve and monitor adherence to interventions

Delivery of all sessions will be underpinned by behaviour change principles to promote adherence to both exercise and nutritional advice and the longer-term maintenance of exercise and a healthy diet. Physiotherapists and dietitians will be trained by a behaviour change scientist in how to promote intrinsic motivation, self-efficacy and habit formation (i.e., key determinants of sustained behaviour change) [38, 39]. In addition, behaviour change strategies will include goal setting, action planning, barrier identification/problem solving, self-monitoring of behavioural outcomes (using a Fitbit activity monitor and goal-setting calendar), relapse prevention/coping planning and providing feedback regarding performance. Participants will be provided with comprehensive and evidence-based behaviour change resources to support self-management as part of the “Physical Activity and Nourishing Eating Toolkit” [40]. This toolkit was reviewed by consumers for participants in the intervention group and, alongside verbal communication, provides clear written communication about all trial interventions.

Furthermore, the intervention is offered using telehealth which allows easier access for rural or working participants and can be planned and timetabled by participants to assist in adherence to attendance. For face to face measurement sessions both parking and coffee vouchers are offered. Exercise intervention sessions may be rescheduled if relevant for the entire group, for example on public holidays. Attendance to intervention sessions, fidelity of intervention delivery and adherence to taking whey protein supplements after exercise sessions will be recorded using a tailored checklist and recorded in a REDCap database. Intervention participants will be defined as adherent to the eight-week 16 session program, if they complete at least 12 sessions (75%) [41]. Session non-attendance due to medical reasons (e.g., hospital admission) will be recorded.

### Relevant concomitant care permitted or prohibited during the trial

All medical and nursing care is permitted throughout the trial. Participants are permitted to conduct their own independent exercise regimens throughout the trial given this is common amongst the general population. Usual care for physiotherapy and nutrition varies across trial sites; however, currently no site provides a verbal or written exercise program as part of usual care at any point in the recovery continuum.

### Outcomes

A summary of outcomes is provided in Table 3.

**Table 3:** Time points and outcomes. Time points measured from baseline (T0) at 30 ± 10 working days post-BMT up to 12 (± 1 m) months (T3) from baseline

Outcome Measure	Screening	Baseline T0 30 (± 10) days post-BMT	T1 Wk 9 (± 2wks)	T2 6 m (± 1 m)	T 3 12 m (± 1 m)
Post-transplant follow-up visit – participant identification	✓				
Participant screening, consent and recruitment	✓				
Demographic, medical and social information		✓			
**Health-related quality of life: Physical function domain of EORTC QLQ-C30 v3**		✓	✓	✓	✓
7-day objective measurement (Fitbit)		✓	✓		
Physical activity: IPAQ-SF		✓	✓	✓	✓
Six-Minute Walk Test		✓	✓		
30 Second Sit to Stand Test		✓	✓		
Body composition:		✓	✓		
Handgrip strength:		✓	✓		
Fatigue: (FACIT-Fatigue)		✓	✓		✓
Sarcopenia risk: SARC-F		✓	✓	✓	✓
Clinical frailty: Clinical Frailty Scale		✓	✓		✓
Dietary intake: three-day food diary		✓	✓		
Malnutrition GLIM and PG-SGA		✓	✓		
Participant acceptability survey: TFA			✓		
Healthcare utilisation			✓	✓	✓
Safety	✓	✓	✓	✓	✓
Survival			✓	✓	✓
Quadriceps point of care ultrasound imaging (in a sub-group)		✓	✓		
Qualitative interviews (in a sub-group)			✓		
Graft versus host disease (Y/N): Assess weekly throughout the trial period					
Process evaluation: measured across intervention and after primary outcome assessments					

*EORTC-QLQ-C30 v3* European Organisation for Research and Treatment of Cancer, Quality of Life Questionnaire version 3, *IPAQ-SF* International physical activity questionnaire -short form, *FACIT-Fatigue* Functional Assessment of Chronic Illness Therapy- fatigue scale, *SARC-F* strength, assistance in walking, rising from a chair, climbing stairs, and falls, *GLIM* Global Leadership on Malnutrition, *PG-SGA* Patient-Generated Subjective Global Assessment, *TFA* Theoretical Framework of Acceptability survey

### Primary outcome

Treatment efficacy will be determined using the primary outcome, physical function, assessed using the physical outcome domain of the European Organisation for Research and Treatment of Cancer core quality of life questionnaire (EORTC QLQ-C30 version 3 [42]) between baseline and nine weeks with the minimal important difference of ≥ 10 points change. This measure is composed of five multi-item domains (physical, role, emotional, cognitive, and social functioning) measured using a four- point Likert scale (Not at all – to Very much) and nine single items. This tool has established validity and reliability in cancer. Permission for use was granted and scoring will follow the EORTC QLQ-C30 version 3 scoring manual guidelines [43]. Participants will complete the entire EORTC QLQ-C30 at baseline and after nine-weeks (follow up 1), 6-months (follow up 2), and 12-months (follow up 3). Participants may complete the questionnaire online, via telephone or in person. Between-group data will be compared. This measure was chosen to align our primary aim to the design of the trial; to improve equity of access for rural and remote participants and since it measures physical function impacting life participation rather than executing a specific activity. Additionally, it allows synthesis of and harmonisation with previous research in cancer rehabilitation [11].

### Secondary outcomes

#### Health-related quality of life

All domains (other than physical function) of role, emotional, cognitive function, and social functioning will be measured using the EORTC QLQ-C30 version 3 [42] and reported as secondary outcomes. It can now also be used to develop a utility measure for *economic outcomes* [44].

#### Fatigue

Functional Assessment of Chronic Illness Therapy – Fatigue Scale (FACIT-Fatigue), which is a 13-item self-reported measure that assesses fatigue and its impact upon activities of daily living. A FACIT-Fatigue score below 34 is considered to be severe and a 10-point improvement a clinically important difference [45–47].

#### Physical function, muscle mass and strength

Functional exercise capacity will be assessed using the six-minute walk test (6MWT) according to standard American Thoracic Society (ATS) guidelines [48]. Physical function and lower limb strength will be assessed using the 30 s sit-to-stand test (*30STS)* using a standardised chair height (43.2 cm) [49]. Handgrip muscle strength will be measured using handgrip dynamometry (Jamar™) tested bilaterally, three measures will be taken on each side following one practice [50]. Data will be presented separately for males and females, right and left sides using the best test and compared between groups as a % change in kg.

#### Physical activity

Objective physical activity including the number of steps taken, cadence, heart rate, as well as time spent in light, moderate, and vigorous intensity zones will be measured using the Fitbit Charge 5 (Fitbit Inc., San Francisco, CA) activity monitor wrist-worn device. Fitbit data will be synced from individual participant Fitbit accounts to Fitabase, a cloud-based data aggregation platform (Fitabase Small Steps Labs, LLC, San Diego, CA) to measure daily activity.

All participants will be instructed to wear their Fitbit during waking hours except for when bathing or swimming for 7-days at baseline and at 9-weeks. One valid day will include a minimum of 8-h of wear time and three valid days will be required to be included in the analysis [51, 52]. Intervention participants will additionally be asked to wear a Fitbit during the intervention period to support physical activity goal setting and monitoring as well as monitoring of heart rate during intervention sessions. They will be able to keep the Fitbit throughout the trial. Usual care participants will only wear the Fitbit at measurement time points and return the Fitbit. Data including steps, cadence and time spent in activity zones will be compared between groups. Physical activity self-report will be obtained using the International Physical Activity Questionnaire – Short Form (IPAQ SF). The IPAQ is the most widely used physical activity assessment method and has established reliability. Although agreement with wearable devices has been low, the IPAQ SF has been used to successfully identify physical activity – health outcome relationships, including those relevant for participants with cancer [53].

#### Muscle size and quality

Rectus femoris muscle cross-sectional area, thickness and echogenicity will be measured using point of care ultrasound (Lumify™, Philips Healthcare) in a sub-group of autologous BMT participants from one site [54]. This sub study will be reported separately.

#### Nutrition

Nutritional status will be assessed using the Patient-Generated Subjective Global Assessment (PG-SGA) and the Global Leadership on Malnutrition criteria (GLIM). The PG-SGA is commonly used tool in clinical practice for the assessment of nutritional status in patients with cancer. The PG-SGA provides a continuous overall score with higher scores indicating higher nutrition risk and a categorical ranking (well-nourished, moderate/suspected malnutrition and severe malnutrition). The PG-SGA has established reliability and validity and has been used to predict clinical outcomes [55, 56].

The *GLIM criteria* are global consensus criteria for the diagnosis of malnutrition [57]. The GLIM criteria consist of three phenotypic criteria (non-volitional weight loss, low body mass index, and reduced muscle mass) and two etiologic criteria (reduced food intake and inflammation) and at least one phenotypic and one etiologic criterion must be met for a diagnosis of malnutrition. The GLIM criteria has established reliability and validity [58].

*Three day food diary:* To determine dietary intake, participants will complete three-day food diaries, with participants instructed to record all food consumed, with approximate food weights or serving sizes, on two weekdays and one weekend day. The three-day recall period is not burdensome to complete and has established reliability [59]. Food diaries will be entered into the Automated Self-Administered 24-h dietary assessment tool (ASA-24-Australia).

**Sarcopenia** risk will be screened using the Strength, Assistance in walking, Rise from a chair, Climb stairs, and Falls (SARC F). This is a self-report questionnaire that contains five items based on features and impacts of sarcopenia each item scored from 0 to 2 with scores ranging from 0–10. A score > 4 is predictive of sarcopenia [60]. Measures of sarcopenia will include low muscle mass using body composition fat-free mass index and reduced muscle strength using hand grip strength results [61].

#### Body composition

Fat-free mass, appendicular lean mass and phase angle will be measured by non-invasive tetrapolar bioimpedance spectroscopy (BIS, SOZO, Impedimed, USA) [62]. Fat-free mass (FFM) will be used to determine the fat-free mass index (FFMI) using the equation FFM (kg)/ height (m^2^). Reduced FFMI is considered < 17 kg/m^2^ in males and < 15 kg/m^2^ in females [63].

**Frailty** will be assessed using the Clinical Frailty Scale (CFS), a clinician assessed rating based on observations of participant mobility, fitness and activity levels, and independence. The CFS is scored out of 7 with a score of 4 indicating living with very mild frailty; 5 mildly frail; 6 moderately and 7 severely frail [64].

#### Demographic, social and medical outcomes

At baseline, demographic and clinical details will be recorded including age, sex, body mass index (BMI), smoking status, medical and social history, cancer diagnosis and stage, treatment details and participant co-morbidities. Survival data will be collected until one-year post study recruitment. Complications will be assessed weekly through the trial period and up to 12 months from medical records, e.g., rate of graft-versus-host disease, and reported descriptively. Reasons for inability to complete intervention or outcome assessments will be recorded.

#### Safety

Adverse events, defined as any unexpected, undesirable event, such as injury, fall, and discomfort related to rehabilitation intervention will be recorded. Adverse events are considered ‘serious’ if they threaten life or function (Common Terminology Criteria for Adverse Events (NCI-CTCAE) v5). All adverse events will be recorded in the trial database, the Chief Investigator notified, attribution specified as well as seriousness of the adverse event. Reporting of any serious adverse events to the trial sponsor and ethics committee will be undertaken by the Chief Investigator within one day of event notification.

### Patient acceptability survey

*Participant acceptability* will be measured using a survey designed for this study based on the Theoretical Framework of Acceptability (TFA) [65, 66].

**Process evaluation** will be conducted in parallel with the RCT to assess three elements, fidelity; causal pathway; contextual influences, based upon the “Framework of Implementability” [67].

- **(a) Intervention fidelity:** We will assess training, delivery enactment and receipt and report findings descriptively including judgements of high, medium or low fidelity using established cut-off scores (80 to 100% adherence to intervention specifications represents ‘high’ fidelity of delivery, 51 to 79% represents ‘moderate’ fidelity, and < 50% or less represents ‘low’ fidelity [68]): (i) *Fidelity of Training* (provider level): Two variables will be assessed: acceptability of the training to providers, measured through a 7-item questionnaire based on Theoretical Framework of Acceptability and provider confidence (‘self-efficacy’) to deliver the intervention in accordance with Standard Operating Procedures (SOPs); (ii) *Fidelity of Delivery* (provider level): The number (%) of SOP-specified intervention components delivered to intervention participants, assessed by checklist, completed by providers after each session, this measurement strategy will be validated by analysing an audio-recording of two sessions per provider each year; (iii) *Fidelity of Receipt* (participant level): Number of sessions attended will be compared with number of sessions planned and on which modes (e.g., physical, nutritional) are offered; iv) Fidelity of Enactment (participant level): Physical activity data, food diary; records of goal setting and action plans.
- **(b) Causal pathway:** We propose that the REBOOT intervention will improve self-reported physical function by ameliorating deconditioning and malnutrition as assessed after the 8-week rehabilitation intervention. These proposed mediating variables will be entered into a series of regression models controlling for values at baseline, to identify whether the intervention has its effect through these causal pathways. Hence, it will be possible to identify whether any, some, or all mediating variables are key to the causal pathway that explains the primary outcome (EORTC QLQ-C30 function domain).
- **(c) Contextual influences:** Mechanisms of change about how the REBOOT intervention interacts with the context will be identified and evaluated to understand how this intervention works in practice. A qualitative exploration of barriers and enablers to participant engagement will be conducted through tele-interviews. A pragmatic target sample of 20 trial participants (10 in the intervention arm; 10 in the usual care arm, with both autologous and allogeneic graft recipients) will be interviewed after the follow-up assessment (9-weeks). It is likely that data saturation will be achieved with this sample size [69]. Interview guides will vary for intervention and usual care groups.

#### Health service use and costs

Healthcare utilisation will be assessed as a composite outcome (GP visits, ambulance callouts, emergency department visits, hospital admissions, hospital length of stay, hospital re-admissions and re-admission length of stay, personal care and domestic support). These data will be obtained using a specific questionnaire developed by the health economist as well as accessing hospital medical records and measured at 9 weeks, 6 and 12 months. Hospital admission costs will be based on the current national average cost for an Australian public hospital separation [70]. Hospital administrative records will be used to supplement resource use data from surveys, developed for the trial. The cost of implementing the program will be estimated from records of staff time taken for service delivery and travel costs. National average hospital award wages for respective staff will be used to estimate the cost-of-service delivery. Travel costs will be estimated based on time taken on a home-visit and distance travelled. Other intervention costs include wearables and protein drink provided.

#### Trial oversight

REBOOT governance structure includes an advisory committee chaired by the Chief Investigator, composing all grant chief investigators and REBOOT trial managers that meets twice per year; and an independent data monitoring committee comprising an independent statistician, haematologist and rehabilitation physiotherapist (Chair) that meets twice yearly. If any important protocol modifications are required following these meetings, the Chief Investigator will request relevant amendments to the trial sponsor and approving ethics committee (Peter MacCallum Human Research Ethics) and make amendments to the trial registry. A steering group chaired by the trial managers and comprising site intervention staff that meets weekly. Each of the three sites has designated site leads.

The protocol was designed with consumer (JW) contributions to protocol development, in particular to intervention and outcome assessments and manuscript preparation.

#### Sample size

We plan to recruit 170 participants in total (85 participants per arm), giving us 80% power at 5% significance using mixed repeated measures ANOVA regression to detect a 10-point difference in the physical function scale of the EORTC-QLQ-C30 v3, with a SD of 20 points. This allows for 25% loss to follow-up: a conservative estimate. The sample size calculation was based on detecting a small between-group effect size. According to Cocks et al. [71] the range of mean differences for a small effect size in the physical function domain is a score of 5–14 and we have chosen the mid-point of this range; 10. A pilot trial by Hung et al., conducted in a similar Australian population lends pilot data support to the choice of a between-group difference of 10 points for the EORTC QLQ-C30 physical functioning scale [72]. This sample is feasible since the three recruitment sites perform 310 transplants/year of approximately equal numbers of BMT types (allogeneic and autologous) and previous recruitment rates (at two Victorian centres) were 93% (allogeneic) and 73% (autologous) of eligible patients [73, 74]. Our team are highly experienced at recruiting, measuring and managing patients with cancer and in conducting rehabilitation.

#### Data management and statistical analyses

A unique study code will be assigned to all participants. All data will be entered into the secure online data management system, REDCap (REDCap, Vanderbuilt University, Nashville USA) database. The database will be accessible only to the research team. Data entry integrity audits will be undertaken by a research team member twice yearly to check for missing and erroneous entries.

All statistical analyses will follow intention-to-treat principles. The trial statistician has developed a full statistical analysis plan, with complete details (attached as Supplementary File 1). A summary is presented here. Descriptive statistics (including counts and percentages for nominal and ordinal variables; and means and standard deviations or medians and interquartile ranges, as appropriate, for continuous variables) will be used to summarise demographic and clinical characteristics. Counts and percentages will be used to summarise data on adverse events (tabulated by severity grade).

Patient-reported outcome measures will be scored according to published recommendations. Means, standard deviations and ranges will be used to summarise continuous outcomes at each time-point by trial arm. Counts and percentages will be used to summarise binary outcomes at each time-point by trial arm. Differences in mean change in baseline-to-9-week physical function score will be compared between the intervention and usual care arms using an ANCOVA regression model, a repeat measures ANOVA, a Linear Mixed Model (LMM) or Generalise Estimating Equations (GEE) as appropriate. The final selection of model or models for the primary outcome will be determined on initial review of the available data.

Kaplan–Meier methods will be used to estimate survival curves with Cox proportional hazards regression models used for time-to-event analysis.

Counts and percentages will be used to summarise missing data, including missing items and forms for patient reported outcome measures. Multiple imputation may be considered as a contingency for missing data in key outcome or explanatory variables, if indicated based on consideration of the variables missing data, the amount of missing data and the pattern of missingness (missing at random vs not-at-random). The selection of explanatory variables for any multiple imputation model will be determined upon review of the available data. Analysis including imputed data will be presented as a sensitivity analysis only and not as part of the primary analysis.

An exploratory subgroup analysis of participants previously exposed to the usual care prehabilitation intervention at Peter MacCallum Cancer Centre will be undertaken. A test of interaction will be used to test for subgroup effects.

Interview guides, within the process evaluation, will be based on the Theoretical Domains Framework (TDF) of behaviour change [75] and will explore the widest possible range of potential barriers, with a particular focus on contextual factors. Findings may include contextual factors that are key for participants living outside urban areas or in economically deprived areas and will likely include factors such as internet availability, living alone, social support networks, physical constraints such as space (for exercise), and availability of healthy foods. Hence, this sub-study aims to uncover specific challenges relating to equity of access that may help to explain variations in engagement and thus intervention effects. Regarding the transcripts, thematic analysis will be performed [76]. Coding and generation of themes using the TDF will be undertaken by two experienced members of the research team. This sub-study will be reported according to COREQ guidelines [77].

#### Economic analysis

A cost analysis and economic evaluation will be conducted from the health care perspective to demonstrate the value of the intervention compared to usual care. Quality Adjusted Life Years (QALYs) will be calculated from the EORTC-QLQ-C30 responses reflecting Australian population norms [78]. An incremental cost-effectiveness ratio (ICER) will be calculated by dividing the difference in total costs (incremental cost) by the difference in health outcome (incremental effect). This will provide a summary measure of the economic value of the proposed intervention compared with the current standard of care. Sensitivity analyses will be conducted to capture uncertainty and to test the robustness of the cost-effectiveness results. Reporting of results will follow the standard economic evaluation methods as outlined in the Consolidated Health Economic Evaluation Reporting Standards statement [79]. The health economic analysis run alongside the clinical trial will be published in a separate paper.

## Discussion

Prior interventional studies of exercise in patients with haematological cancers following stem cell transplant report no serious adverse events, improved exercise capacity and global HRQoL (moderate-certainty evidence) and reduced hospital length of stay (low-certainty evidence) compared to usual care or other interventions [11]. However, serious flaws exist in the design or methodological quality of previous studies of rehabilitation for people with haematological cancers, which are frequently at serious risk of bias and include studies with non-randomised designs, exercising control groups, limited follow-up and small sample sizes [11]. Additionally, most are now over 10 years old and report on only allogeneic BMT [80–82]. Given the different symptom profile and recovery trajectory between allogeneic and autologous transplants, including both in our study provides a unique population. More recent research examined the cardiovascular and exercise limitation impact of allogeneic BMT in a small sample of 17 patients compared with 10 healthy controls. Findings support the reduction in exercise capacity with a reduced V0_2_ peak and the need for exercise intervention in these patients [83]. These authors have also published a protocol to examine exercise and reduced sedentary time in 60 patients during and after allogeneic BMT (ALLO-Active trial) [84]. Their study aims to examine multiple cardiac and physiological exercise intervention end points from a hospital based 12 week thrice weekly exercise intervention compared with usual care.

In contrast, the REBOOT trial will be the first trial to test the effectiveness of a multi-disciplinary tele-rehabilitation program (exercise and physical activity, nutritional counselling, protein supplements and embedded behaviour change techniques) for people who have received allogeneic or autologous BMT for haematological cancer. In keeping with the remote access approach of the trial the primary outcome is a patient-reported measure of physical function.

Several strengths of our trial are worth noting, including the powered sample size, multi-site participant recruitment involving three sites across two Australian states which increases the generalisability of the findings. Additionally, the planned exploratory subgroup analysis provides the opportunity to evaluate the short and long-term effects of both pre- and rehabilitation in people who have received an autologous BMT. Recruitment at 30 (± 10 days) post-transplant aims to support people in the early stages of recovery to mitigate further decline in physical function and muscle mass and associated reduction in recovery. Longer-term (12-month) follow-up of survival and patient-reported outcomes will assess whether any changes in physical activity, and health behaviours are maintained by participants beyond the eight-week period of intervention. The exercise intervention follows recommended FITT-VP guidelines modified for telehealth delivery and all aspects are recorded. There is currently no specific recommendation regarding the most effective exercise type or intensity in this patient population [11, 85]. Finally, alongside the trial an economic analysis of rehabilitation for people following BMT will be undertaken to provide important findings about the cost-effectiveness of the intervention to support future implementation into usual care. There is a paucity of published cost-effectiveness analyses of exercise interventions in this population hence this aspect fills an important research gap.

Although there is a trend toward cancer exercise studies being delivered in the community, the majority published to date have involved supervised, centre-based exercise programs [86]. New models of care are urgently needed to provide equitable services to the growing number of cancer survivors. The use of telehealth in the REBOOT trial, along with online surveys to assess patient-reported outcomes, allows people living outside metropolitan areas, with a suitable device and internet connectivity, to participate. Similar principles are being applied in the Canadian ‘Exercise for Cancer to Enhance Living Well’ (EXCEL) hybrid effectiveness-implementation study which will recruit 1500 people living with and beyond cancer from rural and remote areas to a 12-week, largely online program of exercise and behaviour change and measure outcomes to 1 year [87].

According to the UK MRC guidance on complex interventions, the intervention being tested in the REBOOT trial is complex [88, 89] because of the: (i) number of interacting components in the multi-disciplinary intervention; (ii) number and difficulty of behaviours required by those delivering or receiving the intervention; (iii) number of groups targeted by the intervention; (iv) number and variability of outcomes; and (v) degree of tailoring of the intervention permitted. Complex intervention methods will be used to evaluate this intervention, including assessment of acceptability to participants and a process evaluation. This involves assessing fidelity (was the intervention delivered and received as planned?), clarifying causal mechanisms (how does it work?) and identifying contextual factors (including barriers and enablers) that may explain variation in outcomes [88, 89]. Process evaluation is important for interpreting trial outcomes. For example, if an intervention is clinically effective but does not work through the proposed causal pathway, further exploration is needed prior to scale up, to understand its active components. This will include investigation of fidelity using the National Institute for Health Behaviour Change Consortium framework, [89] which proposes domains of fidelity including training, delivery, enactment and receipt.

The REBOOT trial will provide RCT-evidence from a rigorously conducted, statistically powered multi-site study on the effects of multi-disciplinary tele-rehabilitation for patients with haematological cancer undergoing both autologous and allogeneic BMT. Cost-effectiveness findings of tele-rehabilitation following BMT will be generated. The detailed process evaluation conducted alongside the trial will aid understanding of factors contributing to the trial findings and help guide the design of future trials and implementation of rehabilitation programs into clinical practice for people with haematological cancers.

### Trial status

This trial is recruiting participants at the time of manuscript submission.

## Supplementary Information

Supplementary Material 1: Table 1: Consensus on Exercise Reporting Template (CERT) – details of reporting of the REBOOT intervention.

Supplementary Material 2: Statistical Analysis Plan. Within Supplementary File 1, Table 1: Process Evaluation – timing and measures used, and analysis plan, to evaluate each construct of the process evaluation embedded in the trial.

Supplementary Material 3: Table 2: SPIRIT checklist.

## Data Availability

The datasets used and/or analyses including statistical code will be available from the corresponding author (l.denehy@unimelb.edu.au) on reasonable request on a case-by-case basis beginning 12 months following publication of results.

## Abbreviations

6MWT: Six-minute walk test
30 STS: 30 second sit to stand test
ANCOVA: Analysis of covariance
ANOVA: Analysis of variance
ASA-24: Automated Self-Administered 24-hour dietary assessment tool
ATS: American Thoracic Society
BIS: Bioimpedance spectroscopy
BMI: Body mass index
BMT: Bone marrow transplantation
BORG RPE: Modified BORG rating of perceived exertion scale
CERT: Consensus on exercise reporting template
CFS: Clinical frailty scale
CONSORT: Consolidated standards of reporting trials
COREQ: Consolidated criteria for reporting qualitative research
ECOG: Eastern Cooperative Oncology Group performance status scale
EORTC QLQ-C30: European Organisation for Research and Treatment of Cancer Quality-of-Life Questionnaire Core 30
FACIT-Fatigue: Functional Assessment of Chronic Illness Therapy – Fatigue scale
FFM: Fat free mass
FFMI: Fat free mass index
FITT-VP: Frequency, Intensity, Time and Type, Volume and Progression
GEE: Generalised Estimating Equations
GLIM: Global Leadership on Malnutrition
HR: Heart rate
HRQoL: Health related quality of life
HSCT: Haematopoietic stem cell transplantation
IPAQ-SF: International physical activity questionnaire – short form
IQR: Interquartile range
LMM: Linear mixed models
n: Sample size
NCI-CTCAE v5: National Cancer Institute Common Terminology Criteria for Adverse Events version 5
PA: Physical activity
PG-SGA: Patient- generated-subjective global assessment
PICF: Patient Information and Consent Form
QALYs: Quality Adjusted Life Years
RCT: Randomised controlled trial
REBOOT: Rehabilitation to improve outcomes following bone marrow transplant (short title for this trial)
REDCap: Research Electronic Data Capture
SARC-F: Strength, assistance in walking, rising from a chair, climbing stairs, and falls
SD: Standard deviation
SOP: Standard operating procedure
SPIRIT: Standard Protocol Items: Recommendations for Interventional Trials (2022 Guidelines)
T0: Baseline time point
T1: 9 week assessment time point
T2: 6 month assessment time point
T3: 12 month assessment time point
TDF: Theoretical Domains Framework
TFA: Theoretical Framework of Acceptability
UK MRC: United Kingdom Medical Research Council
