# Supplementary material for: Rehabilitation after bone marrow transplant compared with usual care to improve patient outcomes (REBOOT): protocol for a randomised controlled trial
Source: BMC Cancer. 2025 Mar 24;25:532. doi: 10.1186/s12885-025-13898-3 (PMC11931774; doi:10.1186/s12885-025-13898-3)
Supplement: Supplementary file 2 — Supplementary Material 2: Statistical Analysis Plan. Within Supplementary File 1, Table 1: Process Evaluation – timing and measures used, and analysis plan, to evaluate each construct of the process evaluation embedded in the trial. [file 12885_2025_13898_MOESM2_ESM.docx]

**Supplementary File 1: Statistical Analysis Plan**

**REBOOT – STATISTICAL ANALYSIS PLAN**

**RE**habilitation after **BO**ne marr**O**w **T**ransplant compared with usual care to improve patient outcomes (**REBOOT**): Protocol for a randomised controlled trial

Short title: REBOOT

| **Protocol Number** | 1.0 |
| --- | --- |
| **Country** | Australia |
| **Sponsor** | Clinical trials governance, The University of Melbourne |
| **Coordinating Principal Investigator (CPI)** | Professor Linda Denehy  Department of Physiotherapy, The University of Melbourne, 161 Barry Street, Parkville Victoria 3010, Australia  Department of Health Services Research, Peter MacCallum Cancer Centre, 305 Grattan Street, Melbourne Victoria 3000, Australia |
| **Co-investigators** | Chief Investigators  Prof Linda Denehy  Dr Lara Edbrooke  A Prof Vin Cavalheri  A Prof Nicole Kiss  A Prof Camille Short  Prof Catherine Granger  Associate Investigators  A Prof Amit Khot  Dr Duncan Purtill |
| **SAP author** | Associate Professor Tim Spelman  Department of Health Services Research, Peter MacCallum Cancer Centre, 305 Grattan Street, Melbourne Victoria 3000, Australia |

**Date:** 18^th^ October 2024

**Version:** 1.4

**REVISION HISTORY**

**Revision Date Author(s) Description**

**1.0** 07-08-2024 TS Draft created.

1.1 12-08-2024 TS Draft modified

1.2 17-09-2024 LD Draft modified

1.3 17-10-2024 TS Draft modified

1.4 18-10-2024 LD/LE Final version accepted

Contents

[**1. Abbreviations** 5](#_Toc180156422)

[**2. Introduction** 6](#_Toc180156423)

[2.1 Background and Rationale 6](#_Toc180156424)

[2.2 Intervention 7](#_Toc180156425)

[2.3 Objectives 7](#_Toc180156426)

[2.3.1 Primary objectives 8](#_Toc180156427)

[2.3.2 Secondary objectives 8](#_Toc180156428)

[2.3.3 Exploratory objectives 9](#_Toc180156429)

[**3. Study Design** 9](#_Toc180156430)

[3.1 Type 9](#_Toc180156431)

[3.2 Outcomes 9](#_Toc180156432)

[3.2.1 Primary efficacy outcome 9](#_Toc180156433)

[3.2.2 Secondary outcomes 10](#_Toc180156434)

[3.2.3 Exploratory outcomes 14](#_Toc180156435)

[3.2.4 Safety outcomes 15](#_Toc180156436)

[3.2.5 Cost effectiveness 15](#_Toc180156437)

[3.3 Sample size 16](#_Toc180156438)

[**4. Study population** 16](#_Toc180156439)

[4.1 Eligibility criteria 16](#_Toc180156440)

[4.1.1 Inclusion criteria 16](#_Toc180156441)

[4.1.2 Exclusion criteria 16](#_Toc180156442)

[4.1.3 Additional inclusion criteria 17](#_Toc180156443)

[4.2 Analysis datasets 17](#_Toc180156444)

[**5. Statistical Methods** 17](#_Toc180156445)

[5.1 Data 17](#_Toc180156446)

[5.2 Descriptive analysis 18](#_Toc180156447)

[5.3 Analysis of the primary outcome 18](#_Toc180156448)

[5.3.1 Analysis of covariance (ANCOVA) 18](#_Toc180156449)

[5.3.2 Repeated measures Analysis of Variance ANOVA 19](#_Toc180156450)

[5.3.3 Linear mixed models 19](#_Toc180156451)

[5.3.4 Generalised Estimating Equations 20](#_Toc180156452)

[5.4 Sensitivity analysis of the primary outcome 21](#_Toc180156453)

[5.5 Statistical quantities 21](#_Toc180156454)

[5.6 Analysis of the secondary outcomes 22](#_Toc180156455)

[***5.6.1*** Emotional domain of the ***EORTC QLQ-C30 v3 between baseline and nine weeks & trend over all timepoints*** 22](#_Toc180156456)

[***5.6.2*** Cognitive function domain of the ***EORTC QLQ-C30 v3 between baseline and nine weeks & trend over all timepoints*** 22](#_Toc180156457)

[***5.6.3*** Social functioning domain of the ***EORTC QLQ-C30 v3 between baseline and nine weeks & trend over all timepoints*** 23](#_Toc180156458)

[***5.6.4 Patient reported physical activity*** 23](#_Toc180156459)

[***5.6.5 Functional Assessment of Chronic Illness Therapy – Fatigue Scale (FACIT-Fatigue)*** 23](#_Toc180156460)

[***5.6.6 Six-minute walk test (6MWT)*** 24](#_Toc180156461)

[***5.6.7 30 second sit-to-stand test (30STS)*** 24](#_Toc180156462)

[***5.6.8 Handgrip muscle strength*** 25](#_Toc180156463)

[***5.6.9 Objective physical activity*** 25](#_Toc180156464)

[***5.6.10 Nutritional status*** 26](#_Toc180156465)

[***5.6.11 Sarcopenia: SARC-F*** 26](#_Toc180156466)

[***5.6.12 Body composition*** 27](#_Toc180156467)

[***5.6.13 Frailty***  27](#_Toc180156468)

[***5.6.14 Medical complications (e.g. graft-versus-host disease)*** 28](#_Toc180156469)

[**5.6.15 Survival** 28](#_Toc180156470)

[5.7Analysis of exploratory outcomes 28](#_Toc180156471)

[***5.7.1 Muscle size & quality (sub-study only)*** 28](#_Toc180156472)

[***5.7.2 Process evaluation*** 28](#_Toc180156473)

[5.8 Safety outcomes 31](#_Toc180156474)

[5.9 Cost effectiveness 31](#_Toc180156475)

[5.10 Subgroup analyses 32](#_Toc180156476)

[5.12 Missing data 33](#_Toc180156477)

[5.12.1 Multiple imputation by chained equations (MICE) 33](#_Toc180156478)

[5.12.2 Assessing validity of imputed data 34](#_Toc180156479)

[5.13 Software 34](#_Toc180156480)

[**References** 34](#_Toc180156481)

##

## **1. Abbreviations**

| 30STS | 30 second sit-to-stand test |
| --- | --- |
| 6MWT | Six-minute Walk Test |
| AE | Adverse Event |
| AHPEQS | Australian Hospital Patient Experience Question Set |
| ANOVA | Analysis of Variance |
| ANCOVA | Analysis of Covariance |
| BMT | Bone Marrow Transplantation |
| CFS | Clinical Frailty Score |
| CI | Confidence Interval |
| EORTC QLQ-C30 v3 | European Organisation for the Research and Treatment of Cancer questionnaire version 3 |
| FACIT-Fatigue | Functional Assessment of Chronic Illness Therapy – Fatigue Scale |
| FFM | Fat-free mass |
| FFMI | Fat-free mass index |
| GEE | Generalised Estimating Equations |
| GLIM | Global Leadership Initiative on Malnutrition Criteria |
| HR | Hazard Ratio |
| HRQoL | Health-related Quality of Life |
| HSCT | Haematopoietic Stem Cell Transplantation |
| IQR | Interquartile range |
| LMM | Linear Mixed Models |
| MICE | Multiple Imputation with Chained Equations |
| PG-SGA | Patient-Generated Subjective Global Assessment |
| QIC | Quasi-Likelihood Information Criterion |
| RCT | Randomised Controlled Trial |
| REBOOT | REhabilitation after BOne marrOw Transplant to improve patient outcomes |
| RR | Relative Risk |
| SAE | Serious Adverse Event |
| SARC F | Strength, Assistance in walking, Rise from a chair, Climb stairs, and Falls |
| SD | Standard deviation |
| SE | Standard Error |

##

## **2. Introduction**

The purpose of this document is to describe the statistical methods that will be used to analyse data in the REBOOT multi-site, parallel group, two-arm, randomised controlled superiority trial. It is written for statisticians and should be read in conjunction with the REBOOT trial protocol. The basic study design, structure, objectives, outcomes, endpoints and quantities of interest are outlined and detailed in Section 3, the study population including eligibility and inclusion criteria are described in Section 4 and the statistical methods and models are detailed in Section 5.

## 2.1 Background and Rationale

Haematological cancer accounted for almost 800,000 deaths globally in 2020. In Australia this incidence has risen by nearly 50% over the past 10 years and expected to rise further by 2035. Haematopoietic stem cell transplantation (HSCT), also called bone marrow transplantation (BMT), is a treatment that is commonly used for patients with haematological cancer in an attempt to achieve long-term disease response. However, the intensity of this treatment results in considerable adverse effects including deleterious impacts on nutritional status, physical functioning and overall health-related quality of life.

The REhabilitation after BOne marrOw Transplant to improve patient outcomes (REBOOT) trial is the first multi-site randomised controlled trial using a remote intervention that is adequately powered to test the effectiveness of multidisciplinary tele-rehabilitation including nutrition, exercise, physical activity and behaviour change following allogeneic or autologous BMT. These are crucial factors, as previous research has been underpowered or used unimodal interventions. The overarching aim of the REBOOT trial is to investigate the effectiveness of an eight-week multidisciplinary rehabilitation intervention (nutrition, exercise, physical activity and behaviour change) compared with usual care on both clinical and patient reported outcomes. For a more detailed description of both the study background and rationale, please refer to the accompanying REBOOT trial protocol.

## **2.2 Intervention**

The trial intervention consists of an eight-week multidisciplinary rehabilitation intervention incorporating nutrition, exercise, physical activity and behaviour change which will be provided in addition to usual care. The goal of the intervention is to improve aerobic fitness, muscle mass, nutritional status and strength and facilitate increased physical activity at home following transplant relative to a usual care only comparator. The intervention will be delivered remotely via videoconferencing. For more detail on the various components of the intervention, refer to the accompanying REBOOT trial protocol.

## 2.3 Objectives

The aim of the study is to compare effectiveness of an eight-week multidisciplinary rehabilitation intervention in improving physical function, physical activity, nutritional status, clinical frailty, sarcopenia and body composition, muscle strength, fatigue, health service use and costs, global HRQoL and survival relative to usual care only. Specific objectives are listed in sections 2.3.1, 2.3.2 and 2.3.3 below. The related outcomes are described in section 3.2.

### 2.3.1 Primary objectives

To investigate the effectiveness of an eight-week multidisciplinary rehabilitation intervention (nutrition, exercise, physical activity and behaviour change) on the physical function domain of the European Organisation for the Research and Treatment of Cancer version 3 (EORTC QLQ-C30 v3), health-related quality of life (HRQoL) questionnaire in patients with haematological cancer following bone marrow transplant (allogeneic or autologous), compared to usual care. The primary hypothesis is that the intervention will be effective in improving self-reported physical function at nine weeks post recruitment compared to usual care.

### 2.3.2 Secondary objectives

The secondary objectives of this study are to investigate the effectiveness of the intervention on the following outcomes and endpoints:

1) physical activity levels and objective physical function

2) nutritional status, clinical frailty, sarcopenia, and body composition

3) muscle strength

4) fatigue

5) health service use and costs

6) global HRQoL and emotional, cognitive and social functioning domains of HRQoL

7) survival

### 2.3.3 Exploratory objectives

The exploratory objectives of this study are as follows:

**1)** to determine the impact of treatment on rectus femoris muscle cross sectional area and echogenicity (muscle quality) using point of care muscle ultrasound in a sub-set of participants at a single centre,

**2)** to examine the impact on outcomes in a subset of participants who received both prehabilitation (as part of usual care at one centre) and the trial rehabilitation intervention,

**3)** to conduct a process evaluation exploring fidelity, causal pathways and contextual influences.

## **3. Study Design**

## 3.1 Type

REBOOT is a multi-site, parallel group, two-arm, randomised controlled superiority trial measuring intervention efficacy.

## 3.2 Outcomes

For details on how each outcome is measured and quantified, refer outcome section of the accompanying REBOOT study protocol.

### 3.2.1 Primary efficacy outcome

The primary outcome is the change in the physical functioning domain score of the EORTC QLQ-C30 v3 between baseline and nine weeks. The physical functioning domain of the EORTC QLQ-C30 instrument consists of five items, ranked on a Likert scale from 1 (“Not At All”) to 4 (“Very Much”). The overall total physical function score is derived by using the 5 items in the equation: score = raw score-1/range*.* If at least half the scores from the scale are available all the items that were completed will be used for calculating the scale scores; if half items not recorded then the scale score is reported as missing(1).

Change in this total physical functioning score will be defined as:


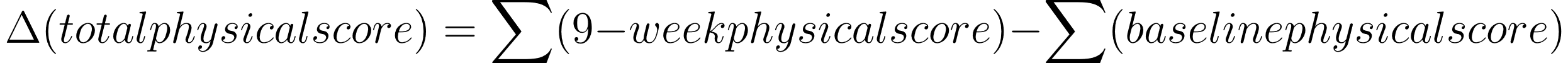


All scales and single-item measures of the questionnaire range from scores of 0-100, with higher scores representing improved HRQoL.

### 3.2.2 Secondary outcomes

**Patient reported outcomes**

1) Global HRQoL scores of the EORTC QLQ-C30 v3 between baseline and nine weeks and differences across time points to 12 months

2) Emotional domain of the EORTC QLQ-C30 v3 between baseline and nine weeks and differences across time points to 12 months

3) Cognitive function domain of the EORTC QLQ-C30 v3 between baseline and nine weeks and differences across time points to 12 months

4) Social functioning domain of the EORTC QLQ-C30 v3 between baseline and nine weeks and differences across time points to 12 months

5) Functional Assessment of Chronic Illness Therapy – Fatigue Scale (version 4) (FACIT-Fatigue): All 13 individual items are summed, multiplied by 13 then divided by the number of items answered to give the fatigue subscale scores. Scores range from 0-52 with higher scores representing lower levels of fatigue. The FACIT-Fatigue is an acceptable indicator of fatigue if overall item response rate is > 80%.

6) Patient reported physical activity: Analysis of the International Physical Activity Questionnaire-Short Form (IPAQ-SF). The scoring for this will follow the algorithms described in the “Guidelines for Data Processing and Analysis of the International Physical Activity Questionnaire (IPAQ) - Short and Long Forms (November 2005)”. The variables derived from the questionnaire are a continuous score, measured in MET-minutes (MET = Metabolic Equivalent of Task, or metabolic equivalent), and a categorical score, measured as one of three levels, “low”, “medium” or “high”. The items making up the scores ask for a number of days during the last 7 days that a defined level of activity was carried out, and the amount of time usually spent doing the activity. The responses for the amount of time usually spent on one of those days have ‘don’t know/not sure’ as an option. If this response has been used, or if data are otherwise missing for time or days, the participant’s response will be taken as missing for this outcome. For available data, comparisons will be made between patient-reported (IPAQ-SF) and objectively measured (Fitbit) physical activity at baseline, 9 weeks and 6 and 12 months.

7) Sarcopenia as measured by the Strength, Assistance in walking, Rise from a chair, Climb stairs, and Falls history (SARC-F). The SARC-F is a rapid screening tool for sarcopenia in older adults. Sarcopenia is defined as a decline in muscle mass, strength and/or function(2). The SARC-F assesses five components including strength, assistance in walking, ability to rise from a chair, ability to climb stairs and occurrence of falls in the past year. The five components are scored from 0 to 2 and combined to derive a total SARC-F score ranging from 0 to 10, with a score ≥4 predictive of sarcopenia.

8) Frailty as measured by the Clinical Frailty Score (CFS). This is a clinician scored document that scores participants based on observations of their mobility, fitness and activity levels, and independence. There are nine possible scores, ranging from very fit (1) to terminally ill (9). Ordinal scores of four or greater identify patients with frailty: Level 4 Living with Very Mild Frailty; Level 5 – Living with Mild Frailty; Level 6 – Living with Moderate Frailty; Level 7 – Living with Severe Frailty. The CFS is a valid and reliable instrument for the detection of frailty, especially among older or hospital-based patients(3).

**Objective Outcomes**

9) Six-minute walk test (6MWT)

Where two 6MWT are performed, two separate distances are recorded in the standard protocol of this test. The larger of the two distances walked (m) of the two tests performed will be used in analyses.

10) 30 second sit-to-stand test (30STS): The number of stands in 30 seconds are recorded. If the patient is over halfway to a standing position when 30 seconds have elapsed, it is counted as a stand.

11) Handgrip muscle strength: Three consecutive efforts are made with a rest in between each repetition on both sides. All three efforts are recorded and reported in kilograms (kg). The highest value of the three tests and left or right sides will be used in analyses.

12) Objective physical activity (Fitbit wrist worn activity monitor device) as measured by: Number of steps recorded, cadence, heart rate, consecutive walking time on a continuous scale). Time spent in light, moderate, and vigorous intensity zones (ordinal scale).The minimum data requirement is three days of eight hours monitoring; otherwise, these measures will be regarded as missing.

13) Nutritional status as measured by:

Patient-Generated Subjective Global Assessment (PG-SGA). The PG-SGA is a common nutrition assessment tool used by oncology dietitians in clinical practice which gives a continuous score as well and categorising patients as: A—well-nourished, B—mild to moderate malnutrition, C—severe malnutrition. The higher the score, the higher the malnutrition risk. The PG-SGA has been evaluated as an outcome measure in clinical nutrition studies and validated for use in oncology patients undergoing radiotherapy. Data collected will also allow determination of a diagnosis of malnutrition using the Global Leadership Initiative on Malnutrition criteria (GLIM).

Global Leadership Initiative on Malnutrition criteria (GLIM): Provides a malnutrition score based upon three phenotypic and two etiological scores as well as severity grading of moderate or severe; both giving categorical scaling.

14) Body composition: Tetrapolar bioimpedance spectroscopy (SOZO, Impedimed, USA) is used to estimate total body and appendicular (arms and legs) fat-free mass and fat mass (all in kg), total body water, extracellular and intracellular fluid and phase angle (ratio of resistance to reactance) using proprietary software provided by Impedimed (Brisbane, Australia). Participants are asked to stand on the SOZO scale, placing feet and hands on the corresponding foot and hand sensors.

15) Medical complications (e.g. graft-versus-host disease) will be recorded descriptively.

16) Survival will be recorded from the medical record at 9-weeks and 6 and censored 12-months.

### 3.2.3 Exploratory outcomes

1) Muscle size & quality (sub-study only)

Rectus femoris is a thigh muscle important for physical functioning. A marker of overall muscle mass and strength, rectus femoris cross-sectional area (RF CSA), measured by ultrasound, can identify patients with muscle wasting. We will measure RF CSA, thickness and echogenicity (muscle fibre quality) at baseline, 9 weeks and 6 months in an outpatient setting using the Lumify™ (Phillips Healthcare) portable ultrasound machine. A linear transducer in B/2D-mode will be used with the minimal probe compression approach at the anterior surface of the thigh, at ½ and 2/3 distance from anterior-superior iliac spine to superior border of the patella, with the patient positioned in supine on a plinth, legs relaxed, straight and neutral. Three images will be taken at each distance on each thigh (12 images total) and will be downloaded in DICOM image format for measurement using DICOM image analysis software. These measures will be compared with tests already being performed as part of the REBOOT trial at the same three time points. Feasibility and acceptability will be reported using descriptive methods.

2) The impact on functional outcomes in a subset of participants who received both prehabilitation (as part of usual care at Peter MacCallum Cancer Centre) and the trial rehabilitation intervention will be examined (refer to section 5.10).

### 3.2.4 Safety outcomes

Adverse events, defined as any unexpected, undesirable event, such as injury, fall, and discomfort related to the rehabilitation intervention will be recorded. Adverse events are considered ‘serious’ if they threaten life or function (Common Terminology Criteria for Adverse Events (CTC AE) version 5 (NCI-CTCAE) v5). All adverse events will be recorded in the trial database, the Chief Investigator notified, attribution specified as well as seriousness of the adverse event. Reporting of any serious adverse events to the trial sponsor and ethics committee will be undertaken by the Chief Investigator within one day of event notification.

### 3.2.5 Cost effectiveness

**Health service use and costs:** Healthcare utilisation will be assessed as a composite outcome (GP visits, ambulance callouts, emergency department visits, hospital admissions, hospital length of stay, hospital re-admissions and re-admission length of stay, personal care and domestic support). These data will be obtained using a specific questionnaire developed by the health economist as well as accessing hospital medical records and measured at 9 weeks, 6 and 12 months. Hospital admission costs will be based on the current national average cost for an Australian public hospital separation(4). Hospital administrative records will be used to supplement resource use data from surveys, developed for the trial. The cost of implementing the program will be estimated from records of staff time taken for service delivery and travel costs. National average hospital award wages for respective staff will be used to estimate the cost-of-service delivery. Travel costs will be estimated based on time taken on a home-visit and distance travelled. Other intervention costs include wearables and protein drink provided.

## 3.3 Sample size

Based on precedent data described in the sample size section of the accompanying REBOOT study protocol, a sample size of 85 participants per arm (total of n=170 participants based on a 1:1 allocation ration) was estimated to have 80% power at the 5% significance level to detect a minimum 10-point difference in the change in the physical functioning domain of the EORTC QLQ-C30 v3 from baseline and nine weeks between the intervention and comparator arms, factoring in 25% loss-to follow-up.

## **4. Study population**

All patients with haematological cancer attending participating centres for bone marrow transplantation.

## 4.1 Eligibility criteria

### 4.1.1 Inclusion criteria

- ≥ 18 years of age

- 30 ± 10 working days after allogeneic or autologous bone marrow transplant (BMT) for the treatment of haematological cancer

- Ambulating independently

- Proficient in English to understand testing and training

### 4.1.2 Exclusion criteria

- Concurrent, actively treated other malignancy or history of other malignancy treated within the past year

- Severe or unstable neurological, cardiorespiratory or musculoskeletal disease or mental illness that might compromise ability to perform exercise

- Unstable psychiatric or cognitive disorders

- Eastern Cooperative Oncology Group (ECOG) performance status >2

### 4.1.3 Additional inclusion criteria

Intervention providers will need to have sufficient experience as described in the “Provider inclusion criteria” sub-section of the REBOOT study protocol.

## 4.2 Analysis datasets

The primary analysis dataset will consist of baseline and longitudinal clinical, treatment, assessment, HRQoL, imaging, management data available for each patient. Refer to Table 3 of the REBOOT study protocol for a full description of outcomes and timepoints captured in the longitudinal dataset. The data will be analysed and reported as observed.

## **5. Statistical Methods**

## 5.1 Data

Individual patient-level data will be collected and collated in REDCap by a unique study code.

## 5.2 Descriptive analysis

Categorical variables will be summarised using frequency and percentage and compared using a chi-square test or Fisher’s exact test as appropriate. Continuous variables will be summarised using mean, standard deviation (SD) and standard error (SE) or median and inter-quartile range (IQR) and compared using a t-test of Wilcoxon rank-sum test as appropriate. Rates will be presented as point estimates with associated 95% confidence intervals presuming an underlying Poisson, negative-binomial and/or zero-inflated Poisson distribution as appropriate. Time-to-event data will be visualised using Kaplan-Meier survival and/or failure curves.

## 5.3 Analysis of the primary outcome

Comparison of the change in the physical functioning domain of the EORTC QLQ-C30 v3 between baseline and nine weeks will be analysed using an ANCOVA regression model, a repeat measures ANOVA, a Linear Mixed Model (LMM) or Generalised Estimating Equations (GEE) as appropriate. The final selection of model or models for the primary outcome will be determined on initial review of the available data.

### 5.3.1 Analysis of covariance (ANCOVA)

An ANCOVA will be used to compare 9-week physical functioning domain of the EORTC QLQ-C30 v3 between the intervention and comparator arms, adjusting for baseline physical score. Group differences will be tested by creating an interaction term between comparison group and time. Goodness-of-fit will be determined via analysis of the model coefficient of determination.

### 5.3.2 Repeated measures Analysis of Variance ANOVA

A repeated measures ANOVA will be used to test for differences in the *change* in physical score from baseline at 9-weeks post-baseline. As per the ANCOVA approach described in section 5.2.2, group differences will be tested by creating an interaction term between user group and time.

### 5.3.3 Linear mixed models

Differences in mean change in baseline-to-9-week physical function score between the intervention and usual care arms will be compared between the intervention and usual care arms using a longitudinal generalised mixed effects regression where trial arm, time and a trial arm-by-time interaction will be modelled as fixed effects. Randomisation strata allogeneic or autologous bone marrow transplant type will be included as random effects. These generalised linear mixed-effects models will take the following form:

*yi*∣*b*∼*Distr*(*μi*,*σ*2*wi*)

*g*(*μ*)=*Xβ*+*Zb*+*δ* ,

where for individual study participant ***i***,

- y is an n-by-1 response vector, and y_i_ is its ith element.
- b is the random-effects vector.
- Distr is a specified conditional distribution of y given b.
- μ is the conditional mean of y given b, and μ_i_ is its ith element.
- σ^2^ is the dispersion parameter.
- w is the effective observation weight vector, and w_i_ is the weight for observation i.
- g(μ) is a link function that defines the relationship between the mean response μ and the linear combination of the predictors.
- X is an n-by-p fixed-effects design matrix.
- β is a p-by-1 fixed-effects vector.
- Z is an n-by-q random-effects design matrix.
- b is a q-by-1 random-effects vector.
- δ is a model offset vector.

### 5.3.4 Generalised Estimating Equations

Generalised Estimating Equations (GEE) extend this generalized linear mixed form to further model the covariance structure (the structure describing the correlation between physical function measures per subject). For the primary analysis, the GEE will first be defined using an exchangeable covariance matrix (all physical function scores and other measurements over time per patient have the same correlation). If the exchangeable covariance structure leads to non-convergence, Quasi-Likelihood Information Criterion (QIC) will be used to select the best covariance structure which may include either an autoregressive (first-order) correlation structure (where the correlation between repeated measures decreases as a power of how many timepoints apart two observations are) or an unstructured correlation matrix (correlation is permitted to vary between all pairs of successive timepoints). Model based estimates of the changes from baseline, SE and corresponding 95% confidence intervals (CIs) will also be provided along with p-values for assessing statistical significance, both longitudinally and at each key post-baseline time-point.

The GEE model will include the treatment group, the study visit/time point and an interaction between treatment group and visit/time-point and will take the following generalized linear form:


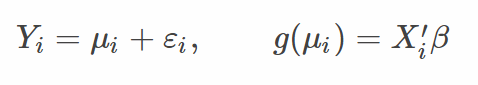


- where for individual ***i***,
- **- Y*_i_*** = outcome change (e.g. physical function score)
- **- X*_i_*** = the model covariates (e.g. intervention vs comparator, any adjusting covariates)
- **- β** = a vector of coefficients
- **- ε_i_**  = random error term
- **- g** = **link function**that maps from the set of possible responses to a linear function of the covariates

## 5.4 Sensitivity analysis of the primary outcome

The subgroup analyses described in section 5.8 below will additionally act as sensitivity analysis of the primary outcome across the full analysis set. Additional sensitivity analyses of the primary outcome will be determined once the initial data review is complete.

## 5.5 Statistical quantities

Comparative effect sizes will be quantified using β-coefficients, hazard ratios (HR), odds ratios (OR) or risk ratios/relative risk (RR) as appropriate. Point estimate effect sizes will be presented with the associated 95% confidence intervals (95% CI). Statistical significance will be taken as a p<0.05.

## 5.6 Analysis of the secondary outcomes

### ***5.6.1*** Emotional domain of the ***EORTC QLQ-C30 v3 between baseline and nine weeks & trend over all timepoints***

Descriptive statistics will be used to summarise the emotional domain of the EORTC QLQ-C30 v3, at both baseline and nine weeks in addition to the change between the two timepoints. Comparison of the change in the emotional domain score between baseline and nine weeks by treatment group will be analysed using an ANCOVA regression model, a repeat measures ANOVA, a Linear Mixed Model (LMM) or Generalised Estimating Equations (GEE) as appropriate and as described above. The final selection of model or models for this outcome will be determined on initial review of the available data.

### ***5.6.2*** Cognitive function domain of the ***EORTC QLQ-C30 v3 between baseline and nine weeks & trend over all timepoints***

Descriptive statistics will be used to summarise the cognitive function domain of the EORTC QLQ-C30 v3, at both baseline and nine weeks in addition to the change between the two timepoints. Comparison of the change in the cognitive function domain score between baseline and nine weeks by treatment group will be analysed using an ANCOVA regression model, a repeat measures ANOVA, a Linear Mixed Model (LMM) or Generalised Estimating Equations (GEE) as appropriate and as described above. The final selection of model or models for this outcome will be determined on initial review of the available data.

### ***5.6.3*** Social functioning domain of the ***EORTC QLQ-C30 v3 between baseline and nine weeks & trend over all timepoints***

Descriptive statistics will be used to summarise the social functioning domain of the EORTC QLQ-C30 v3, at both baseline and nine weeks in addition to the change between the two timepoints. Comparison of the change in the social functioning domain score between baseline and nine weeks by treatment group will be analysed using an ANCOVA regression model, a repeat measures ANOVA, a Linear Mixed Model (LMM) or Generalised Estimating Equations (GEE) as appropriate and as described above. The final selection of model or models for this outcome will be determined on initial review of the available data.

### ***5.6.4 Patient reported physical activity***

Descriptive statistics will be used to summarise IPAQ-SF patient reported physical activity, at both baseline and any post-baseline assessment points in addition to the change between timepoints. Comparison of patient-reported physical activity metrics at single timepoints between treatment groups will be conducted using a t-test or Wilcoxon rank-sum test. Comparison of the change in patient-reported physical activity between baseline and any post-baseline assessment points will be analysed using paired tests such as paired t-test or signed-rank tests, an ANCOVA regression model, a repeat measures ANOVA, a Linear Mixed Model (LMM) or Generalised Estimating Equations (GEE) as appropriate and as described above. The final selection of model or models for this outcome will be determined on initial review of the available data.

### ***5.6.5 Functional Assessment of Chronic Illness Therapy – Fatigue Scale (FACIT-Fatigue)***

Descriptive statistics will be used to summarise FACIT-Fatigue score, at both baseline and any post-baseline assessment points in addition to the change between timepoints. Comparison of FACIT-Fatigue scores at single timepoints between treatment groups will be conducted using a t-test or Wilcoxon rank-sum test. Comparison of the change in the FACIT-Fatigue score between baseline and any post-baseline assessment points will be analysed using paired tests such as paired t-test or signed-rank tests, an ANCOVA regression model, a repeat measures ANOVA, a Linear Mixed Model (LMM) or Generalised Estimating Equations (GEE) as appropriate and as described above. The final selection of model or models will be determined on initial review of the available data.

### ***5.6.6 Six-minute walk test (6MWT)***

Descriptive statistics will be used to summarise 6MWT, at both baseline and any post-baseline assessment points in addition to the change between timepoints. Comparison of 6MWT scores at single timepoints between treatment groups will be conducted using a t-test or Wilcoxon rank-sum test. Comparison of the change in the 6MWT score between baseline and any post-baseline assessment points will be analysed using paired tests such as paired t-test or signed-rank tests, an ANCOVA regression model, a repeat measures ANOVA, a Linear Mixed Model (LMM) or Generalised Estimating Equations (GEE) as appropriate and as described above. The final selection of model or models will be determined on initial review of the available data.

### ***5.6.7 30 second sit-to-stand test (30STS)***

Descriptive statistics will be used to summarise 30STS, at both baseline and any post-baseline assessment points in addition to the change between timepoints. Comparison of 30STS scores at single timepoints between treatment groups will be conducted using a t-test or Wilcoxon rank-sum test. Comparison of the change in the 30STS score between baseline and any post-baseline assessment points will be analysed using paired tests such as paired t-test or signed-rank tests, an ANCOVA regression model, a repeat measures ANOVA, a Linear Mixed Model (LMM) or Generalised Estimating Equations (GEE) as appropriate and as described above. The final selection of model or models will be determined on initial review of the available data.

### ***5.6.8 Handgrip muscle strength***

Descriptive statistics will be used to summarise handgrip muscle strength, at both baseline and any post-baseline assessment points in addition to the change between timepoints. Comparison of muscle strength scores at single timepoints between treatment groups will be conducted using a t-test or Wilcoxon rank-sum test. Comparison of the change in the muscle strength score between baseline and any post-baseline assessment points will be analysed using paired tests such as paired t-test or signed-rank tests, an ANCOVA regression model, a repeat measures ANOVA, a Linear Mixed Model (LMM) or Generalised Estimating Equations (GEE) as appropriate and as described above. The final selection of model or models will be determined on initial review of the available data.

### ***5.6.9 Objective physical activity***

Descriptive statistics will be used to summarise number of steps taken, cadence, consecutive walking time, heart rate and time spent in various intensity zones, at both baseline and any post-baseline assessment points in addition to the change between timepoints. Comparison of these physical activity metrics at single timepoints between treatment groups will be conducted using a t-test or Wilcoxon rank-sum test. Comparison of the change in the activity metrics between baseline and any post-baseline assessment points will be analysed using paired tests such as paired t-test or signed-rank tests, an ANCOVA regression model, a repeat measures ANOVA, a Linear Mixed Model (LMM) or Generalised Estimating Equations (GEE) as appropriate and as described above. The final selection of model or models will be determined on initial review of the available data.

### ***5.6.10 Nutritional status***

Descriptive statistics will be used to summarise PG-SGA and GLIM nutritional status scores, at both baseline and any post-baseline assessment points in addition to the change between timepoints. Comparison of these nutritional status metrics at single timepoints between treatment groups will be conducted using a t-test or Wilcoxon rank-sum test. Comparison of the change in the nutritional status between baseline and any post-baseline assessment points will be analysed using paired tests such as paired t-test or signed-rank tests, an ANCOVA regression model, a repeat measures ANOVA, a Linear Mixed Model (LMM) or Generalised Estimating Equations (GEE) as appropriate and as described above. The final selection of model or models will be determined on initial review of the available data.

### ***5.6.11 Sarcopenia: SARC-F***

Descriptive statistics will be used to summarise Strength, Assistance in walking, Rise from a chair, Climb stairs, and Falls (SARC-F) scores, at both baseline and any post-baseline assessment points in addition to the change between timepoints as both a continuous measure and as a binary outcome where indicated (e.g. overall score ≥ 4). Comparison of these nutritional status metrics at single timepoints between treatment groups will be conducted using a t-test, Wilcoxon rank-sum test or chi-square test as indicated. Comparison of the change in the nutritional status between baseline and any post-baseline assessment points will be analysed using paired tests such as paired t-test, signed-rank tests or McNemar chi-square, an ANCOVA regression model, a repeat measures ANOVA, a Linear Mixed Model (LMM) or Generalised Estimating Equations (GEE) as appropriate and as described above. The final selection of model or models will be determined on initial review of the available data.

### ***5.6.12 Body composition***

Descriptive statistics will be used to summarise fat-free mass (FFM), appendicular lean mass, phase angle and fat-free mass index (FFMI), at both baseline and any post-baseline assessment points in addition to the change between timepoints. Comparison of body composition metrics at single timepoints between treatment groups will be conducted using a t-test, Wilcoxon rank-sum test or chi-square test as indicated. Comparison of the change in body composition between baseline and any post-baseline assessment points will be analysed using paired tests such as paired t-test, signed-rank tests or McNemar chi-square, an ANCOVA regression model, a repeat measures ANOVA, a Linear Mixed Model (LMM) or Generalised Estimating Equations (GEE) as appropriate and as described above. The final selection of model or models will be determined on initial review of the available data

### ***5.6.13 Frailty***

Descriptive statistics will be used to summarise clinical frailty score (CFS) at both baseline and any post-baseline assessment points in addition to the change between timepoints, as both a continuous measure and as a binary outcome where indicated. Comparison of CFS metrics at single timepoints between treatment groups will be conducted using a t-test, Wilcoxon rank-sum test or chi-square test as indicated. Comparison of the change in CFS between baseline and any post-baseline assessment points will be analysed using paired tests such as paired t-test, signed-rank tests or McNemar chi-square, an ANCOVA regression model, a repeat measures ANOVA, a Linear Mixed Model (LMM) or Generalised Estimating Equations (GEE) as appropriate and as described above. The final selection of model or models will be determined on initial review of the available data

### ***5.6.14 Medical complications (e.g. graft-versus-host disease)***

Medical complications will be presented as rates with 95% confidence intervals and compared between treatment groups using a Poisson, negative-binomial or zero-inflated Poisson count model as appropriate.

### **5.6.15 Survival**

Kaplan-Meier estimates, a log-rank test and/or Cox regression will be used to describe and analyse time-to-event.

## 5.7Analysis of exploratory outcomes

### ***5.7.1 Muscle size & quality (sub-study only)***

In the exploratory analysis of patients eligible for this sub-study, descriptive statistics will be used to summarise muscle size and quality at both baseline and any post-baseline assessment points in addition to the change between timepoints. Comparison of size and quality at single timepoints between treatment groups will be conducted using a t-test, Wilcoxon rank-sum test or chi-square test as indicated. Comparison of the change in muscle size and/or quality between baseline and any post-baseline assessment points will be analysed using paired tests such as paired t-test, signed-rank tests or McNemar chi-square, an ANCOVA regression model, a repeat measures ANOVA, a Linear Mixed Model (LMM) or Generalised Estimating Equations (GEE) as appropriate and as described above. The final selection of model or models for the outcome will be determined on initial review of the available data

### ***5.7.2 Process evaluation***

Table 1 outlines the timing and measures used, and analysis plan to evaluate each construct of the process evaluation embedded in the trial. Descriptive statistics will be used to summarise the intervention acceptability and fidelity. Data to explore causal pathways will be analysed using regression models. The final selection of model or models will be determined on initial review of the available data. Contextual influences relating to recruitment sites will be described qualitatively. Interview guides, within the process evaluation, will be based on the Theoretical Domains Framework (TDF) of behaviour change (5) and will explore the widest possible range of potential barriers, with a particular focus on contextual factors. Findings may include contextual factors that are key for participants living outside urban areas or in economically deprived areas and will likely include factors such as internet availability, living alone, social support networks, physical constraints such as space (for exercise), and availability of healthy foods. Hence, this sub-study aims to uncover specific challenges relating to equity of access that may help to explain variations in engagement and thus intervention effects. Regarding the transcripts, thematic analysis will be performed (6). Coding and generation of themes using the TDF will be undertaken by two experienced members of the research team. This sub-study will be reported according to COREQ guidelines (7). Causal mechanisms will be evaluated as part of the outcome measures of the main trial results

**Table 1:** Process Evaluation – timing and measures used, and analysis plan, to evaluate each construct of the process evaluation embedded in the trial.

| **Construct** | **Target** | **Framework** | **Method(s)** | **Analysis** | **Time point** |
| --- | --- | --- | --- | --- | --- |
| **Acceptability**  *Is REBOOT appropriate?* | Recipient  HCPs | TFA(8, 9) | Validated questionnaire | Descriptive | Recipient: Follow-up 1 at 9 weeks  HCPs: at end of trial intervention |
| **Fidelity of provider training**  *Did providers receive training to deliver REBOOT?* | HCPs | TFA(8, 9) | Validated questionnaire | Descriptive | HCPs: at end of trial intervention |
| **Fidelity of intervention delivery**  *Was REBOOT delivered as intended?* | HCPs | NIH BCC(10) | Fidelity checklist; audio / video recordings | Number of SOP-specified intervention components | Completion of each intervention session (twice weekly) |
| **Fidelity of intervention receipt**  *Did participants understand REBOOT?* | Recipient | TFA(8, 9) | Mixed methods: Validated questionnaire; semi-structured interviews | Descriptive | Follow-up 1 at 9 weeks |
| **Fidelity of intervention enactment**  *Did participants apply knowledge and skills from REBOOT in their daily life?* | Recipient | NIH BCC(10) | Mixed methods: Using step count data (Fitbit); food diary. Semi-structured interview | Descriptive | Follow-up 1 at 9 weeks |
| **Causal mechanisms**  *How does REBOOT work?* | Participants | BCTs(5) | Mixed-methods: Semi-structured interviews (10 participants in each group); mediating variables. | Thematic analysis; regression models. | Follow-up |
| **Contextual influences**  *Which contextual factors influence REBOOT?* | Participants  HCPs | CFIR(11) | Key demographic, existing data from sites and from the study database | Descriptive analysis | Follow-up 1 at 9 weeks |
| **Feasibility: Barriers / Enablers**  *What factors influenced uptake of REBOOT?* | Participants  HCPs | TDF(5)  FIM(12) | Participants: Semi-structured interviews (10 participants in each group)  HCPs: validated questionnaire | Thematic analysis  Descriptive | Follow up |

***Abbreviations****: BCTs, behavioural change techniques; CFIR, Consolidated Framework for Implementation Research; FIM, Feasibility of Intervention Measure; HCPs, healthcare practitioners; NIH BCC, National Institute of Health Behaviour Change Consortium;* *SOP, standard operating procedures; TDF, Theoretical Domains Framework; TFA, Theoretical Framework of Acceptability.*

## 5.8 Safety outcomes

Adverse events (AEs) and Serious adverse events (SAEs) will be summarised descriptively as expected or unexpected and their relationship with the intervention will be reported. Safety events will be reported as point estimates with associated 95% confidence intervals using the exact Poisson method.

## 5.9 Cost effectiveness

**Economic analysis:** A cost analysis and economic evaluation will be conducted from the health care perspective to demonstrate the value of the intervention compared to usual care. Generalised linear models (GLM) will be used to analyse costs. The family to distribution to be used will be determined using the modified Park test and its appropriate link determined using Pearson correlation, Pregibon and modified Homer and Lemeshow tests. Important covariates (such as age, sex, type of transplant (allogeneic or autologous) will be included in the models to control for possible baseline imbalances. The choice of models will be based on the Akaike information criterion (AIC). To account for sampling uncertainty, sensitivity analysis will be undertaken using bootstrapping with 1000 replications using the recycled predictions method.

For the economic evaluation, patient-level utility values will be calculated from the EORTC-QLQ-C30 v3 responses reflecting Australian population norms (13). Utility values will be combined with the study period of time to calculate effectiveness in quality adjusted life years (QALYs) terms. Intervention effects with respect to EORTC-QLQ-C30 v3 scores will be estimated using methods specified for the main analysis of patient level clinical outcome measures, controlling for the same set of pre-specified set of potential confounders.

 An incremental cost-effectiveness ratio (ICER) will be calculated by dividing the difference in total costs (incremental cost) by the difference in health outcome (incremental effect).  Results from the economic evaluation will be expressed as cost per QALY gained. This will provide a summary measure of the economic value of the proposed intervention compared with the current standard of care.  Sensitivity analyses will be conducted to capture uncertainty and to test the robustness of the cost-effectiveness results. Reporting of results will follow the standard economic evaluation methods as outlined in the Consolidated Health Economic Evaluation Reporting Standards statement(14). The health economic analysis run alongside the clinical trial will be published in a separate paper.

## 5.10 Subgroup analyses

Subgroup analyses will be performed for the following key sub-populations:

- Participants previously exposed to the usual care prehabilitation intervention at Peter MacCallum Cancer Centre. A test of interaction will be performed to formally test for subgroup effects
- Muscle size & quality at Peter MacCallum Cancer Centre: using point of care ultrasound measuring RF CSA, thickness and echogenicity (muscle fibre quality) using the Lumify™ (Phillips Healthcare) portable ultrasound machine.

The percentage difference in RF CSA between the two groups will be compared using unadjusted and adjusted regression analyses to identify the relationship between RF CSA and the other independent variables (function, sarcopenia, strength, fat-free mass, complications).

## 5.12 Missing data

Multiple imputation of missing data during follow up and prior to censoring may be considered in the following scenarios:

1) ≥ 10% missing data in any key outcome variable

2) ≥ 10% missing data in any key explanatory or confounder variable

### 5.12.1 Multiple imputation by chained equations (MICE)

Where key outcome and/or confounder information is missing, multiple imputation will be used to replace each missing observation with a set of imputed values using the multiple imputation with multivariate imputation by chained equation (MICE) package in R (15, 16). For each variable for which missing data is to be imputed, the missing data will be defined as a function of a set of explanatory covariates determined to be representative of the sample being imputed and for which data is fully available (i.e. no missing data).

### 5.12.2 Assessing validity of imputed data

The validity of the multiply imputed data will be assessed via:

1) Graphical comparisons of imputed and observed values for plausibility, including kernel density plots and histograms (17)

2) Comparison of regression goodness-of-fit of multiply imputed dataset vs original complete-case (non-imputed) model (17, 18)

## 5.13 Software

Data processing, compilation and analysis will be conducted using R (R Foundation for Statistical Computing, Vienna, Austria) and Stata (StataCorp, College Station, Texas).

## **References**

1. Fayers PM AN, Bjordal K, Groenvold M, Curran D, Bottomley A, on behalf of the EORTC Quality of Life Group. The EORTC QLQ-C30 Scoring Manual (3rd Edition). 2001.

2. Malmstrom T, Morley J. SARC-F: a simple questionnaire to rapidly diagnose sarcopenia. (1538-9375 (Electronic)).

3. Rockwood K, Song X, MacKnight C, Bergman H, Hogan DB, McDowell I, et al. A global clinical measure of fitness and frailty in elderly people. Canadian Medical Association Journal. 2005;173(5):489-95.

4. Independent Hospital Pricing Authority. National Hospital Cost Data Collection Cost Report: Round 20 Financial Year 2015-16. Canberra2018.

5. Atkins L, Francis J, Islam R, O'Connor D, Patey A, Ivers N, et al. A guide to using the Theoretical Domains Framework of behaviour change to investigate implementation problems. Implement Sci. 2017;12(1):77.

6. Braun V, Clarke V. Using thematic analysis in psychology. Qualitative Research in Psychology. 2006;3(2):77-101.

7. Tong A, Sainsbury P, Craig J. Consolidated criteria for reporting qualitative research (COREQ): a 32-item checklist for interviews and focus groups. Int J Qual Health Care. 2007;19(6):349-57.

8. Sekhon M, Cartwright M, Francis JJ. Acceptability of healthcare interventions: an overview of reviews and development of a theoretical framework. BMC Health Serv Res. 2017;17(1):88.

9. Sekhon M, Cartwright M, Francis JJ. Development of a theory-informed questionnaire to assess the acceptability of healthcare interventions. 2022(1472-6963 (Electronic)).

10. Bellg AJ, Borrelli B Fau - Resnick B, Resnick B Fau - Hecht J, Hecht J Fau - Minicucci DS, Minicucci Ds Fau - Ory M, Ory M Fau - Ogedegbe G, et al. Enhancing treatment fidelity in health behavior change studies: best practices and recommendations from the NIH Behavior Change Consortium. 2004(0278-6133 (Print)).

11. Damschroder LJ, Reardon CA-O, Widerquist MAO, Lowery J. The updated Consolidated Framework for Implementation Research based on user feedback. 2022(1748-5908 (Electronic)).

12. Weiner BJ, Lewis CC, Stanick C, Powell BJ, Dorsey CN, Clary AS, et al. Psychometric assessment of three newly developed implementation outcome measures. Implementation Science. 2017;12(1):108.

13. King MT, Viney R, Simon Pickard A, Rowen D, Aaronson NK, Brazier JE, et al. Australian Utility Weights for the EORTC QLU-C10D, a Multi-Attribute Utility Instrument Derived from the Cancer-Specific Quality of Life Questionnaire, EORTC QLQ-C30. PharmacoEconomics. 2018;36:225-38.

14. Husereau D, Drummond M, Augustovski F, de Bekker-Grob E, Briggs AH, Carswell C, et al. Consolidated Health Economic Evaluation Reporting Standards 2022 (CHEERS 2022) statement: updated reporting guidance for health economic evaluations. BMC Medicine. 2022;20(1):23.

15. Zhang Z. Multiple imputation with multivariate imputation by chained equation (MICE) package. Ann Transl Med. 2016;4(2):30.

16. Azur MJ, Stuart EA, Frangakis C, Leaf PJ. Multiple imputation by chained equations: what is it and how does it work? Int J Methods Psychiatr Res. 2011;20(1):40-9.

17. Nguyen CD, Carlin JB, Lee KJ. Model checking in multiple imputation: an overview and case study. Emerg Themes Epidemiol. 2017;14:8.

18. Abayomi K, Gelman A, Levy M. Diagnostics for Multivariate Imputations. Journal of the Royal Statistical Society Series C. 2008;57:273-91.
