# Supplementary material for: Rehabilitation after bone marrow transplant compared with usual care to improve patient outcomes (REBOOT): protocol for a randomised controlled trial
Source: BMC Cancer. 2025 Mar 24;25:532. doi: 10.1186/s12885-025-13898-3 (PMC11931774; doi:10.1186/s12885-025-13898-3)
Supplement: Supplementary file 3 — Supplementary Material 3: Table 2: SPIRIT checklist. [file 12885_2025_13898_MOESM3_ESM.docx]

**REBOOT Protocol Paper – Denehy et al., 2024.**

**Supplementary Table 1:** Consensus on Exercise Reporting Template (CERT) – details of reporting of the REBOOT intervention

| **Item category** | **Item No.** | **Abbreviated Item Description** | **Location in REBOOT protocol** |
| --- | --- | --- | --- |
| **WHAT:** materials | 1 | Type of exercise equipment | - Page 13 – **Intervention**, lines 257-258, “…three resistance bands (light, moderate and firm resistance)…” - Page 14 – *Exercise intervention*, line 282-283, “…resistance bands and using body weight…” |
| **WHO:** provider | 2 | Qualifications, teaching/supervising expertise, and/or training of the exercise instructor | - Page 10 – **Provider inclusion criteria**, lines 193-199, “…physiotherapists who are >2 years since graduation and have experience in managing patients with cancer… training for all intervention staff from a behaviour change scientist… recorded videos, face to face demonstration and intervention manuals.” |
| **HOW:** delivery | 3 | Whether the exercises are performed individually or as a group | - Page 13 – *Exercise intervention*, line 267-268, “…group based where possible with up to four participants” |
|  | 4 | Whether the exercises are supervised or unsupervised | - Page 13 – *Exercise intervention*, line 266-267, “…supervised by a physiotherapist…via telehealth.” |
|  | 5 | Measurement and reporting of adherence to exercise | - Page 15 – *Exercise intervention*, line 304-305, “…activity monitor will be provided to intervention participants to track their physical activity…” - Page 18 – **Strategies to improve and monitor adherence to interventions**, line 359-364, “Attendance to intervention sessions, fidelity of intervention delivery and adherence to taking whey protein supplements after exercise sessions will be recorded using a tailored checklist and recorded in a REDCap database…” |
|  | 6 | Details of motivation strategies | - Page 17 – **Strategies to improve and monitor adherence to interventions**, line 342-352, “Delivery of all sessions will be underpinned by behaviour change principles… strategies will include action planning, barrier identification/problem solving, self-monitoring of behavioural outcomes (using a Fitbit activity monitor and goal-setting calendar) … resources to support self-management…” |
|  | 7 | Decision rules for progressing the exercise program | - Page 14 – *Exercise intervention*, line 285-298, “A Traffic light system (Figure 3)…guide intensity… exercise progressed according to current exercise guidelines and participant ability… progressed using modified BORG RPE” - Page 15 – **Table 2:** Exercise Frequency, Intensity, Time, Type, Volume and Progression (FITT-VP) principles used for REBOOT intervention participants |
|  | 8 | Each exercise is described so that it can be replicated (eg, illustrations, photographs) | - Page 14 – E*xercise intervention,* line 288-291, “…individualised… for example marching on the spot… sit to stand…” - Page 15 – **Table 2:** Exercise Frequency, Intensity, Time, Type, Volume and Progression (FITT-VP) principles used for REBOOT intervention participants |
|  | 9 | Content of any home program component | - Page 15 – E*xercise intervention,* line 300-307, “A home program of moderate intensity continuous physical activity will be prescribed aiming to be active on the days not attending group sessions on telehealth…” - Page 15 – **Table 2:** Exercise Frequency, Intensity, Time, Type, Volume and Progression (FITT-VP) principles used for REBOOT intervention participants |
|  | 10 | Non-exercise components | - Page 12-13, **Intervention,** line 252-259, “…nutrition and embedded behaviour change”, “Physical Activity and Nourishing Eating Toolkit”… - Page 14, *Exercise intervention,* line 278-279, “…one-to-one education with the physiotherapist regarding exercise in haematological cancer…” - Page 16-17, **Nutrition,** line 320-327, “…dietary advice to achieve a protein of 1.5g/kg body weight, and 25 to 30 kcal/kg/body weight… participants will also be instructed to take a powdered whey-based protein supplement (20g protein) within 30 minutes of exercise twice weekly…” - Page 18, **Strategies to improve and monitor adherence to interventions,** line 342-352, “Delivery of all sessions will be underpinned by behavour change principles… strategies will include goal setting, action planning, barrier identification/problem solving, self-monitoring of behavioural outcomes…” |
|  | 11 | How adverse events that occur during exercise are documented and managed | - Page 25, **Outcomes – Safety,** line 481-487, “Adverse events… such as injury, fall, and discomfort related to rehabilitation intervention… will be recorded in the trial database, the Chief Investigator notified…” |
| **WHERE:** location | 12 | Setting in which the exercises are performed | - Page 9, **Study design and setting,** line 183, “The intervention will be delivered remotely via videoconferencing.” - Page 13, *Exercise intervention,* line 269-270, “The delivery model will be via telehealth using videoconferencing platforms available within recruitment sites and/or the Zoom platform.” |
| **WHEN, HOW MUCH:** dosage | 13 | Detailed description of the exercises (eg sets, repetitions, duration, intensity) | - Page 14 – *Exercise intervention*, line 282-298, “…aerobic and resistance exercises… intervals of four exercises per set and for three sets…one minute work…30-60 seconds of rest… moderate-high intensity 4-6/10…” - Page 15 – **Table 2:** Exercise Frequency, Intensity, Time, Type, Volume and Progression (FITT-VP) principles used for REBOOT intervention participants |
| **TAILORING:** what, how | 14 | Whether the exercises are generic (“one size fits all”) or tailored to the individual | - Page 14-15 – *Exercise intervention*, line 285, “…will be individualised by the intervention physiotherapist depending upon participant ability…" - Page 15 – *Exercise intervention*, line 302, “…program will be individualised, following the Frequency, Intensity, Time and Type, Volume and Progression (FITT-VP) principles…” |
|  | 15 | Decision rule that determines the starting level of exercise | - Page 14 – *Exercise intervention*, line 292-298, “A traffic light screening system (Figure 3) will be used before each session… to guide exercise intensity and timing of intervals for that individual in the session. Heart rate (measured using a Fitbit) and/or modified BORG RPE will guide exercise intensity…” |
| **HOW WELL:** planned, actual | 16 | Whether the exercise intervention is delivered and performed as planned | - Page 18, **Strategies to improve and monitor adherence to interventions,** line 359-361, “Attendance to intervention sessions, fidelity of intervention delivery and adherence to taking whey protein supplements after exercise sessions will be recorded using a tailored checklist and recorded in a REDCap database…” - Page 26, **Outcomes – Intervention fidelity,** line 501-504, “The number (%) of SOP-specified intervention components delivered to intervention participants, assessed by checklist, completed by providers after each session…” |
